# Supplementary material for: Characterization of genome-wide STR variation in 6487 human genomes
Source: Nat Commun. 2023 Apr 12;14:2092. doi: 10.1038/s41467-023-37690-8 (PMC10097659; doi:10.1038/s41467-023-37690-8)
Supplement: Supplementary file 1 — Supplementary Information [file 41467_2023_37690_MOESM1_ESM.docx]

# Supplementary Materials

Characterization of genome-wide STR variation in 6,487 human genomes


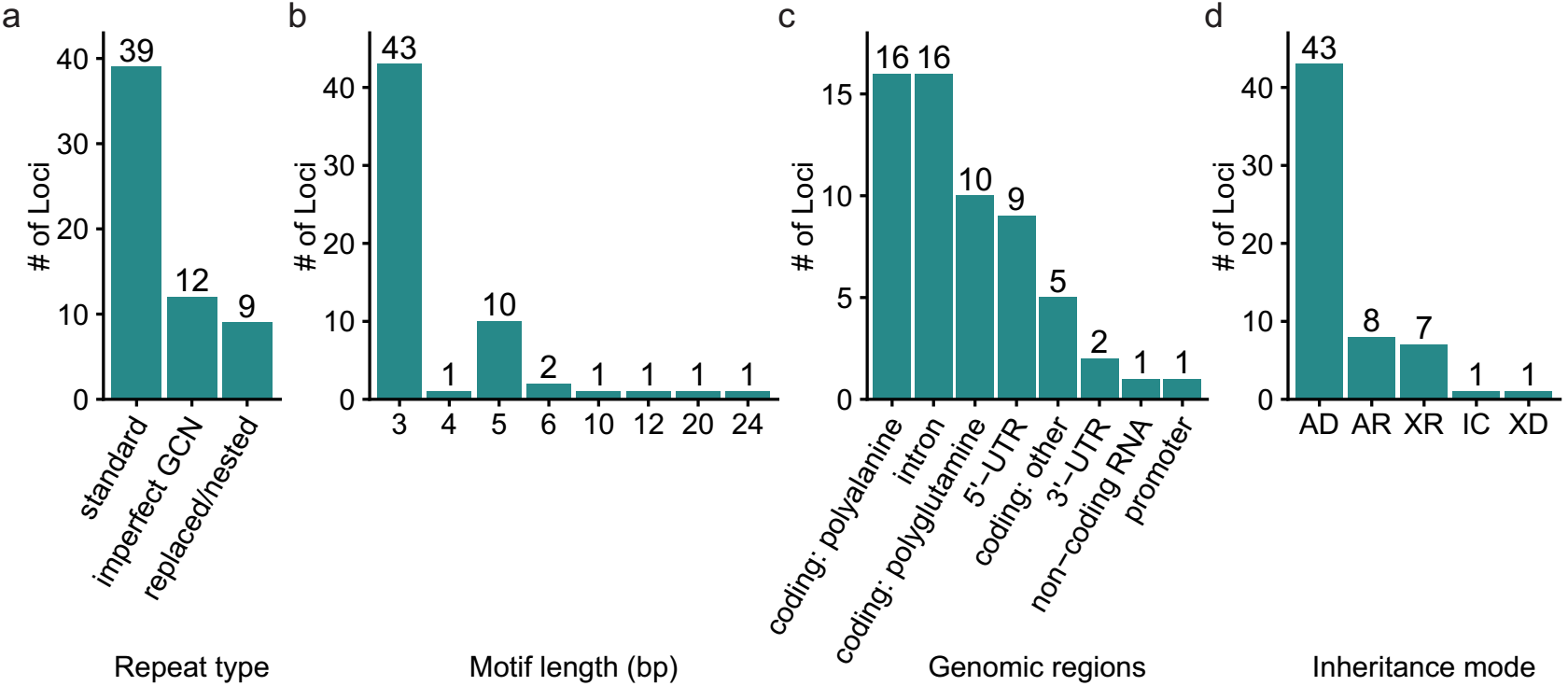


**Fig. S1 Characteristics of known pathogenic STR loci collected in this study**. Distribution of the repeat type (**a**), motif length (**b**), genomic region (**c**), and inheritance mode (**d**) of pathogenic STR loci collected in this study. Inheritance mode: AD, autosomal dominant; AR, autosomal recessive; XR, X-linked recessive; IC, isolated cases; XD, X-linked dominant.


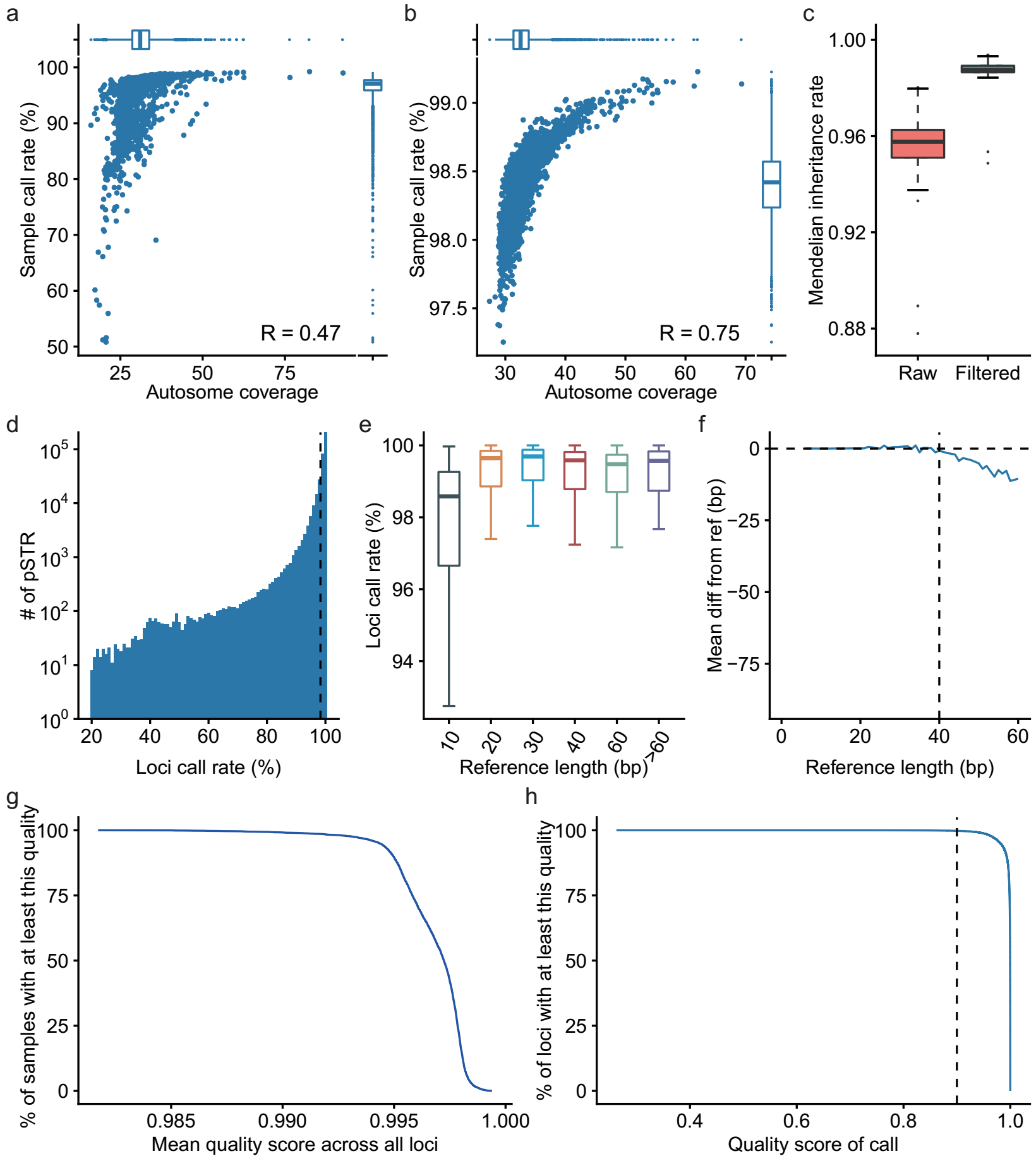


**Fig. S2 Quality control of STR call set. a-b** Correlation between the coverage of autosomes and the STR call rate for each sample of NyuWa (a) and 1KGP dataset (b). The horizontal and vertical boxes show the distribution of x-axis values and y-axis values, respectively. **c** Distribution of mendelian inheritance rates across genome-wide STRs in 60 trios from NyuWa dataset before (Raw) and after filtering (Filtered). Horizontal lines indicate the median and boxes span from the lower quartile (the 25th percentiles) to the upper quartile (the 75th percentiles). Whiskers extend to points that are within 1.5 × IQR (interquartile range) from the upper or the lower quartiles. **d** Distribution of the call rate per pSTR locus. The black dashed line indicates the mean value. **e** Distribution of the loci call rate per pSTR locus (n = 366,013) grouped by motif length in base pairs (bp). Horizontal lines indicate the median and boxes span from the lower quartile (the 25th percentiles) to the upper quartile (the 75th percentiles). Whiskers extend to points that are within 1.5 × IQR (interquartile range) from the upper or the lower quartiles. 10, 0-10 bp; 20, 11-20 bp; 30, 21-30 bp; 40, 31-40 bp; 60, 41-60 bp. **f** Correlations between the mean length difference (base pairs) of all alleles and the reference allele length for each pSTR locus. **g-h** The cumulative distribution of the mean quality scores across all STR loci for each sample (g) or mean quality scores across all calls for each STR locus (h).


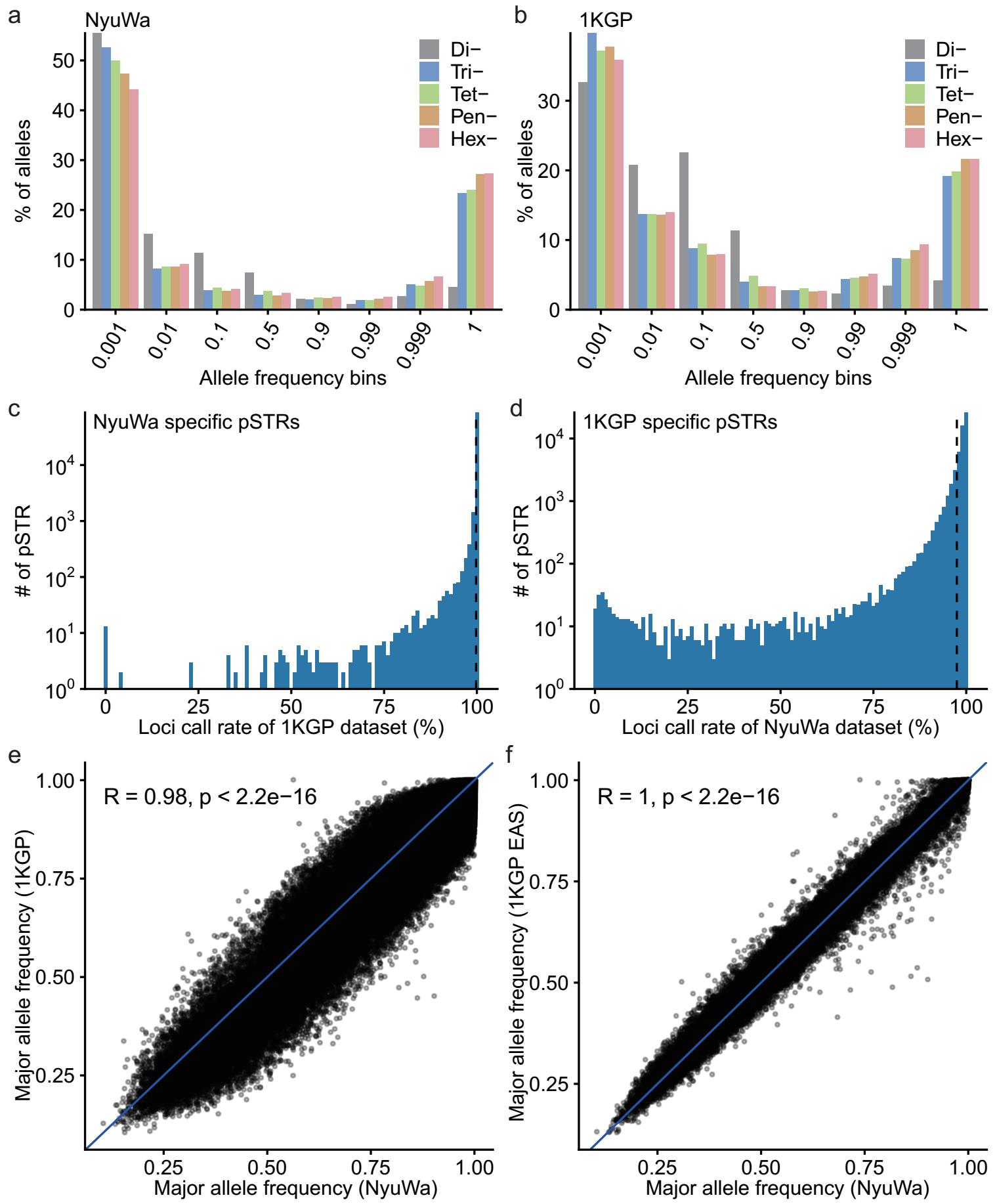


**Fig. S3 Comparison of pSTR call sets identified from NyuWa and 1KGP dataset. a-b** Distribution of allele frequencies (AF) of all pSTR alleles stratified by motif length in NyuWa (a) and 1KGP (b) dataset. The allele frequencies were cut into eight bins: 0 ≤ AF < 0.001, 0.001 ≤ AF < 0.01, 0.01 ≤ AF < 0.1, 0.1 ≤ AF < 0.5, 0.5 ≤ AF < 0.9, 0.9 ≤ AF < 0.99, 0.99 ≤ AF < 0.999 and 0.999 ≤ AF < 1. **c** Distribution of loci call rate of NyuWa specific pSTRs in 1KGP dataset. **d** Distribution of locus call rate of 1KGP specific pSTRs in NyuWa dataset. **e-f** Correlation plot between the allele frequencies of the major allele (aka. most common allele) of pSTR loci generated from NyuWa dataset and that generated from the 1KGP dataset (e) and East Asian (EAS) samples in the 1KGP dataset (f). Pearson correlation coefficients and the corresponding P-values (two-sided *t*-test) were shown.


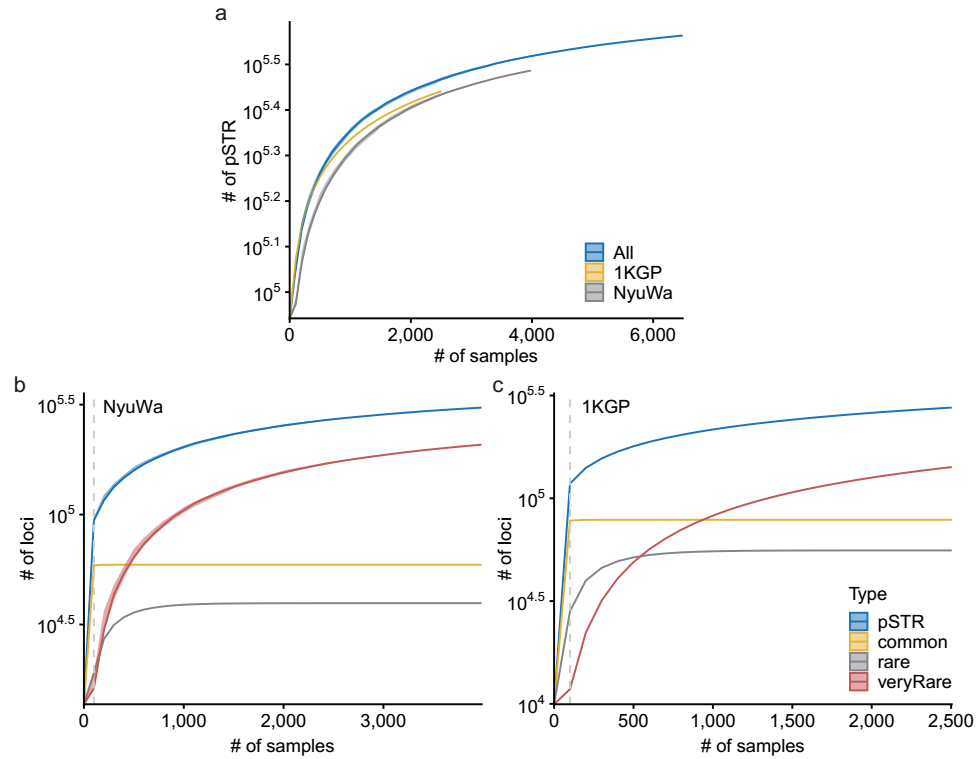


**Fig. S4 Saturation curves for our pSTR call set. a** The saturation analysis of pSTR loci discovered in all samples, in the NyuWa samples, and in the 1KGP samples. **b-c** The saturation analysis of pSTR loci discovered in NyuWa (b) and 1KGP (c) datasets classified by pSTR allele frequencies. pSTR, all pSTR loci; common, minor allele frequency ≥ 0.01; rare, 0.001 ≤ minor allele frequency < 0.01; veryRare, minor allele frequency < 0.001.


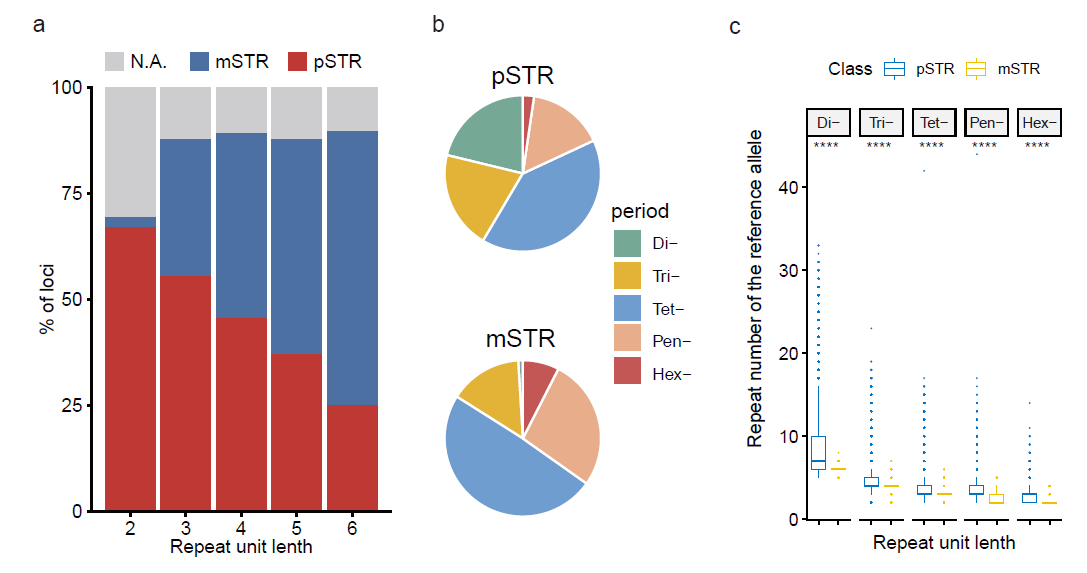


**Fig. S5 pSTRs and mSTRs in our STR call set. a** The proportion of different types of STR loci. N.A. indicates the STRs that were in the reference file of GangSTR but were filtered in our STR call set. **b** The number of pSTRs (upper) or mSTRs (lower) with different motif lengths. **c** Distribution of the repeat number of the reference alleles for pSTR (n = 366,013) or mSTR (n = 290,454) with different motif lengths. Horizontal lines indicate the median and boxes span from the lower quartile (the 25th percentiles) to the upper quartile (the 75th percentiles). Whiskers extend to points that are within 1.5 × IQR (interquartile range) from the upper or the lower quartiles.


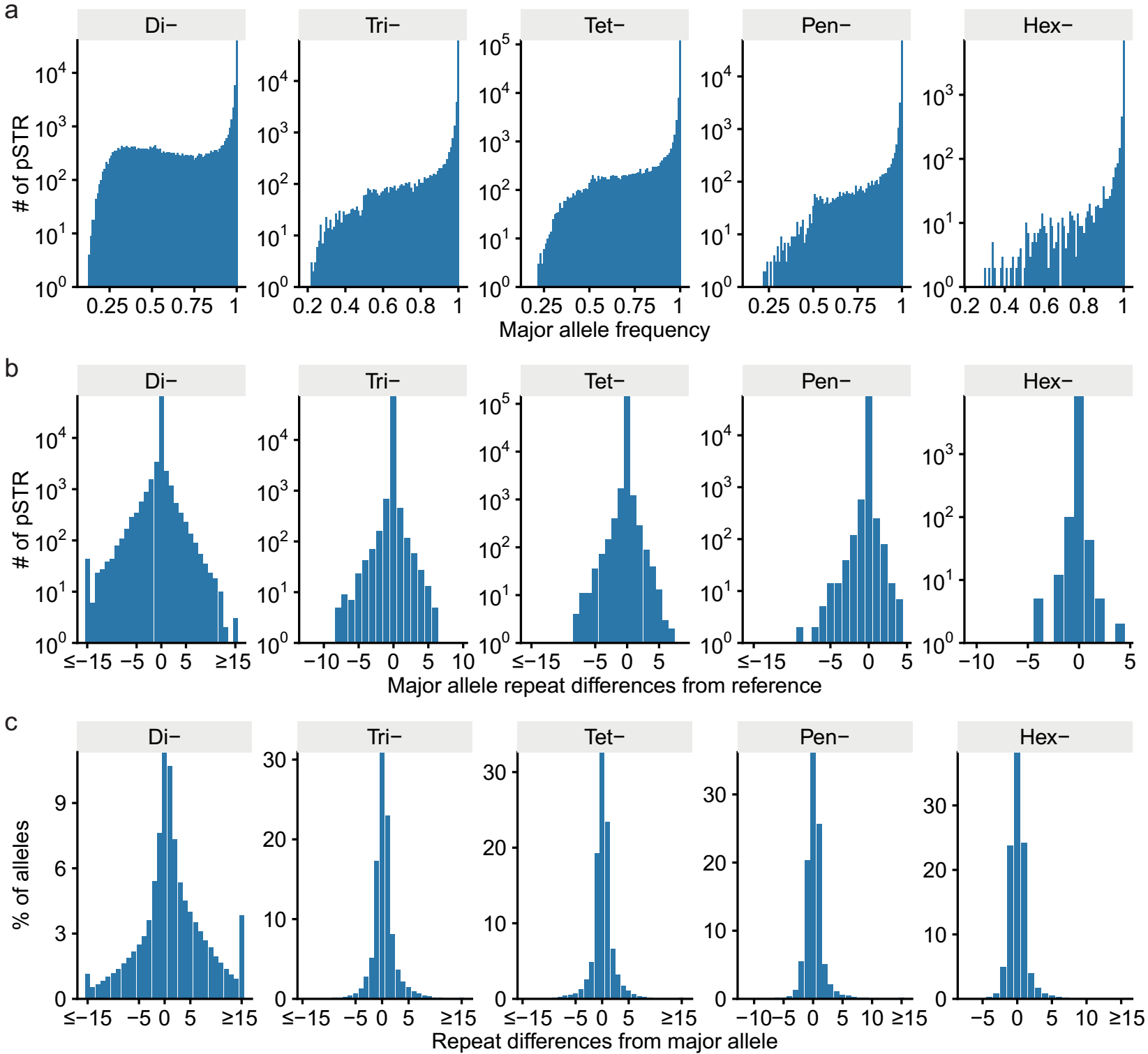


**Fig. S6 pSTR mutational patterns by motif length. a** Distribution of major allele frequencies for pSTR loci stratified by motif length. **b** Distribution of the difference of repeat numbers between major allele and reference allele for all pSTR loci stratified by motif length. **c** Distribution of the difference of repeat numbers between pSTR allele and the major allele of the corresponding locus stratified by motif length.

**
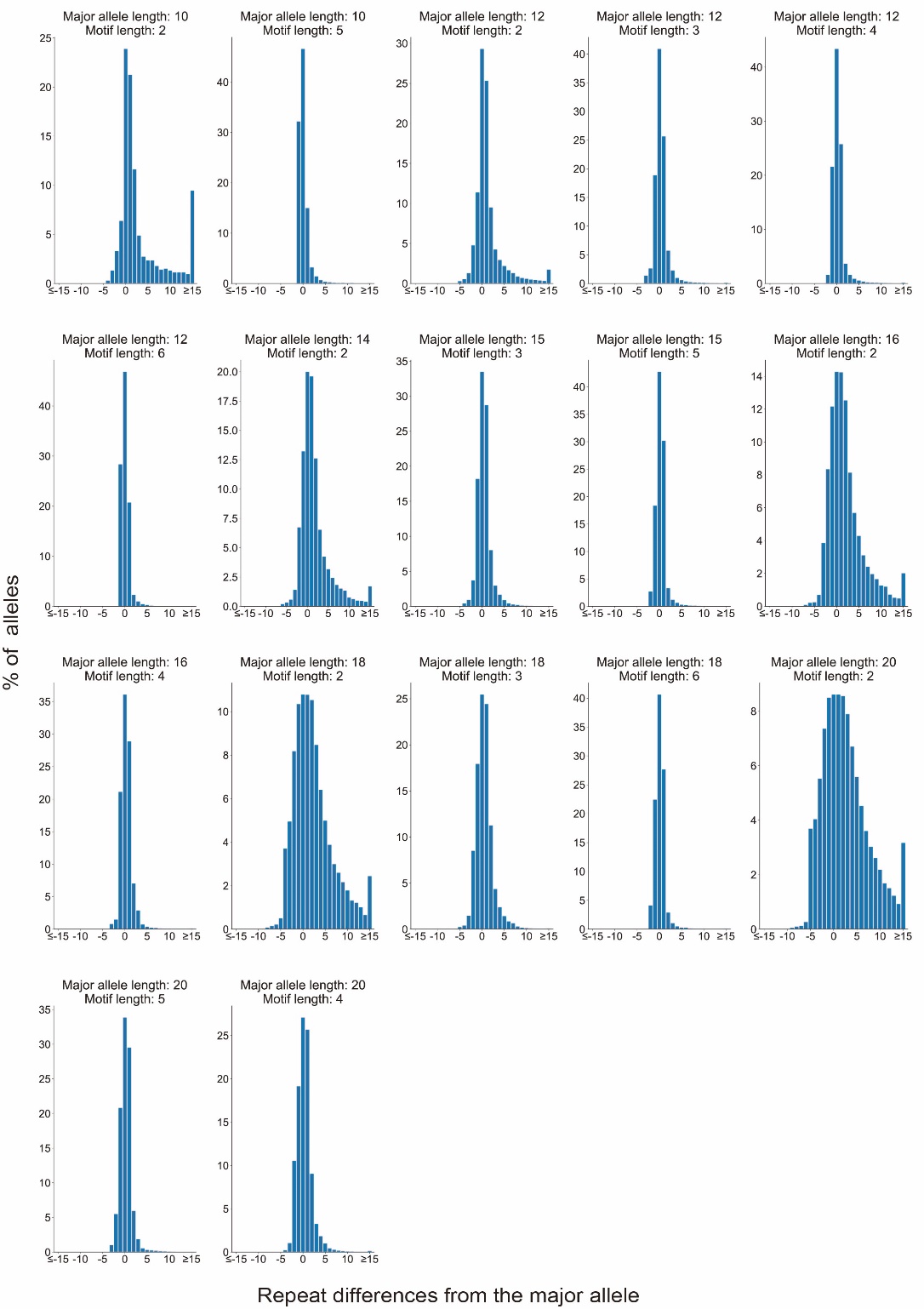
**

**Fig. S7 Distribution of the difference of repeat numbers between pSTR allele and the major allele of the corresponding locus stratified by motif length and major allele length.** Only pSTRs with major allele lengths between 10 and 20 bp were displayed.


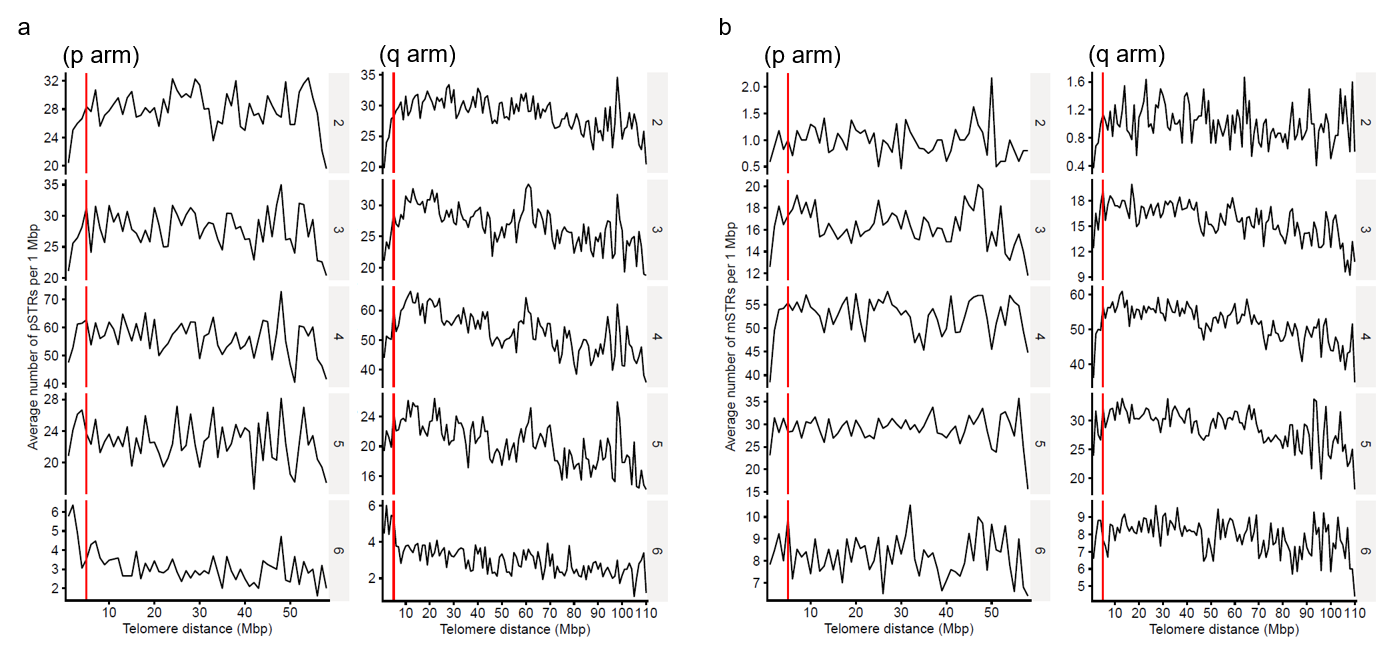


**Fig. S8 Distribution of STR loci near telomeres.** The number of pSTRs (**a**) and mSTRs (**b**) in each 1 Mbp bin as a function of telomere distance, for p arms and q arms, respectively, with STRs stratified by motif length. The red lines indicate 5 Mbp to the telomeres.


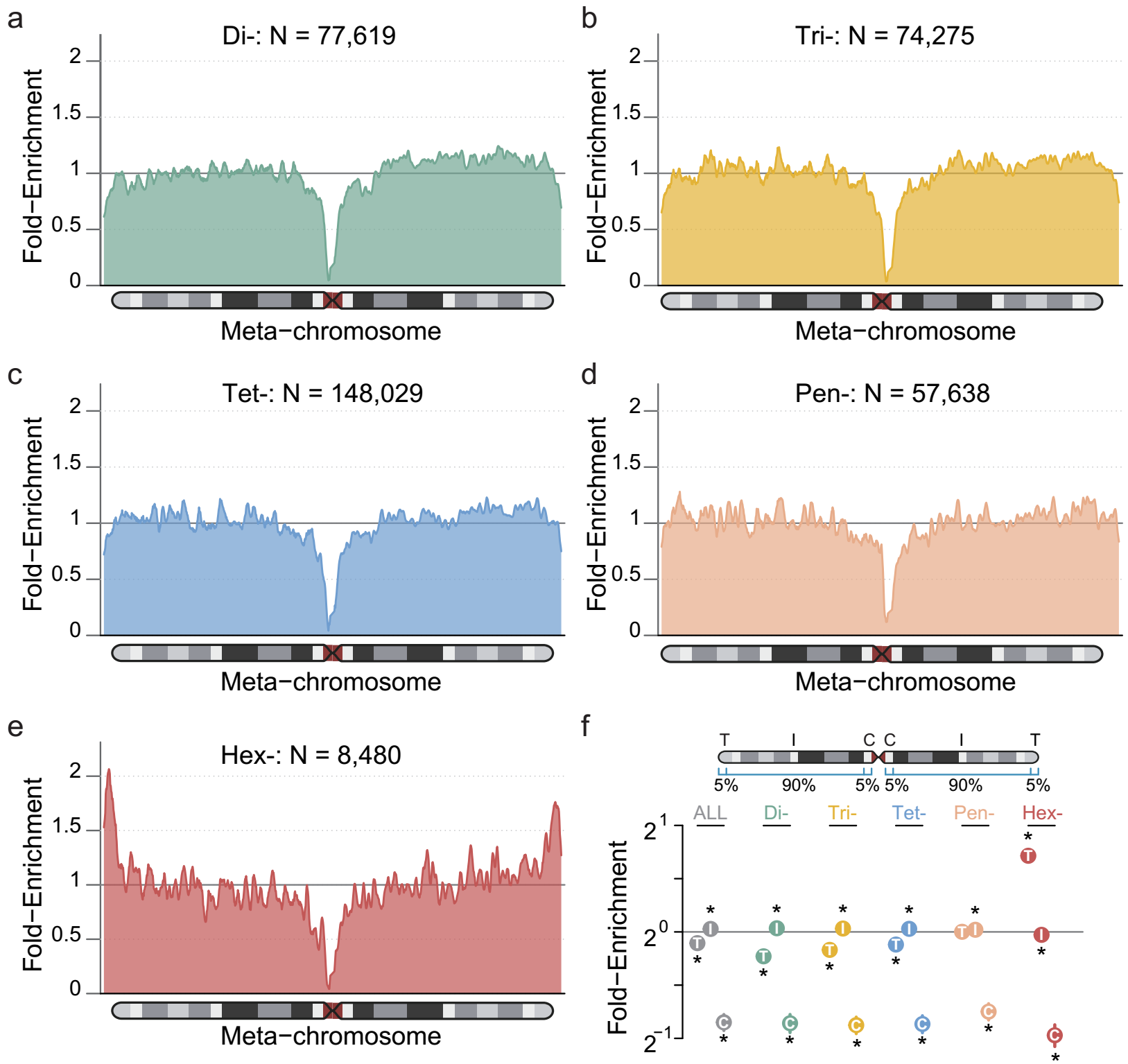


**Fig. S9 Chromosome-level distribution of pSTR density. a-e** Smoothed enrichment of pSTRs with motif lengths of 2 (a), 3 (b), 4 (c), 5 (d) and 6 (e) bp, respectively. The values were calculated per 100 kb window across the average of all autosomes and normalized by the length of chromosome arms (as ‘meta-chromosome’). **f** Enrichment of pSTRs by motif length and chromosomal context. The dots indicate the mean values. P-values were computed using student’s t-test and adjusted using the Bonferroni method. *, P-value ≤ 0.05. C, centromeric; I, interstitial; T, telomeric.


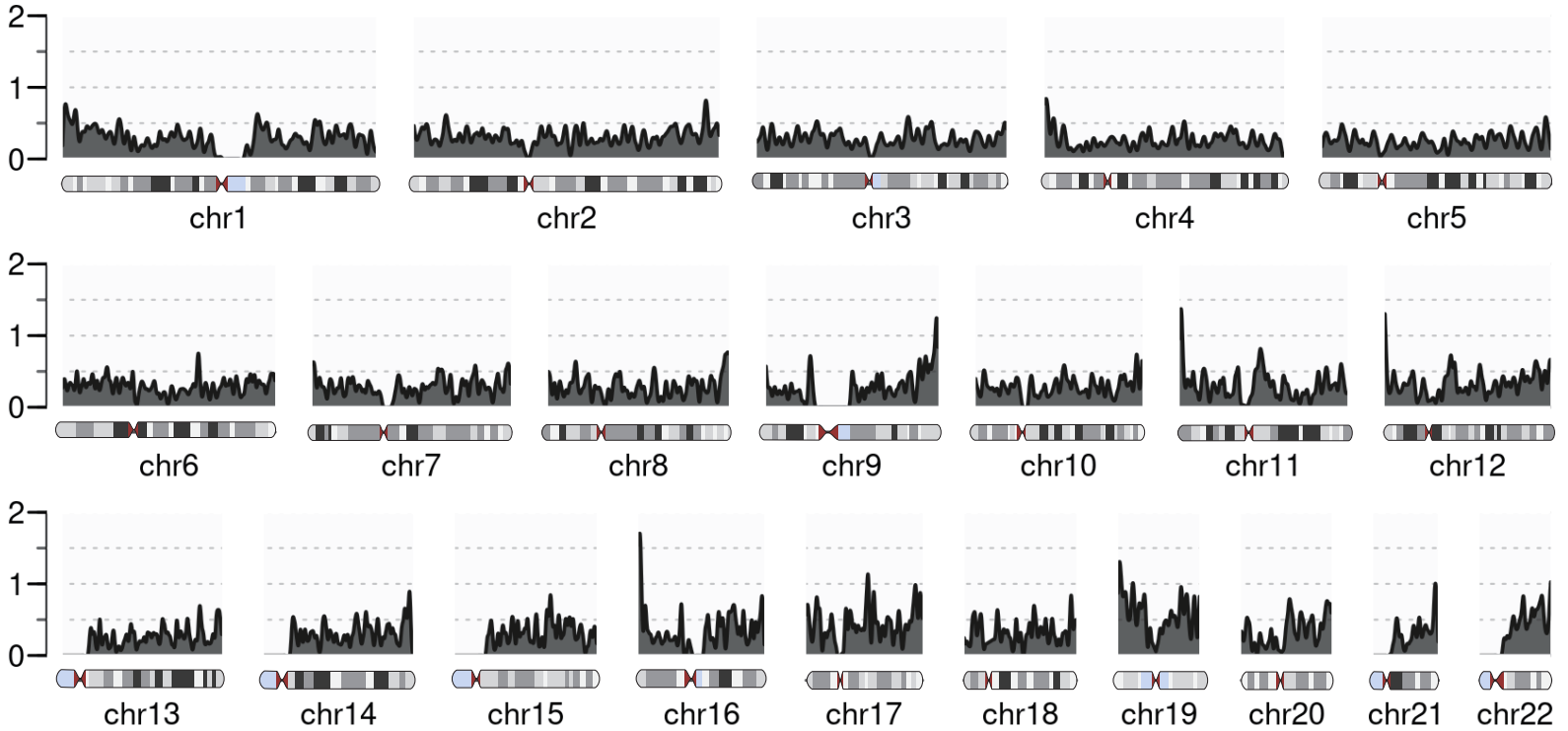


**Fig. S10 Smoothed enrichment of hexameric pSTRs per chromosome.** The density of hexameric pSTRs was computed per autosome in 100 kb sequential windows, represented here as a 1 Mbp rolling mean.


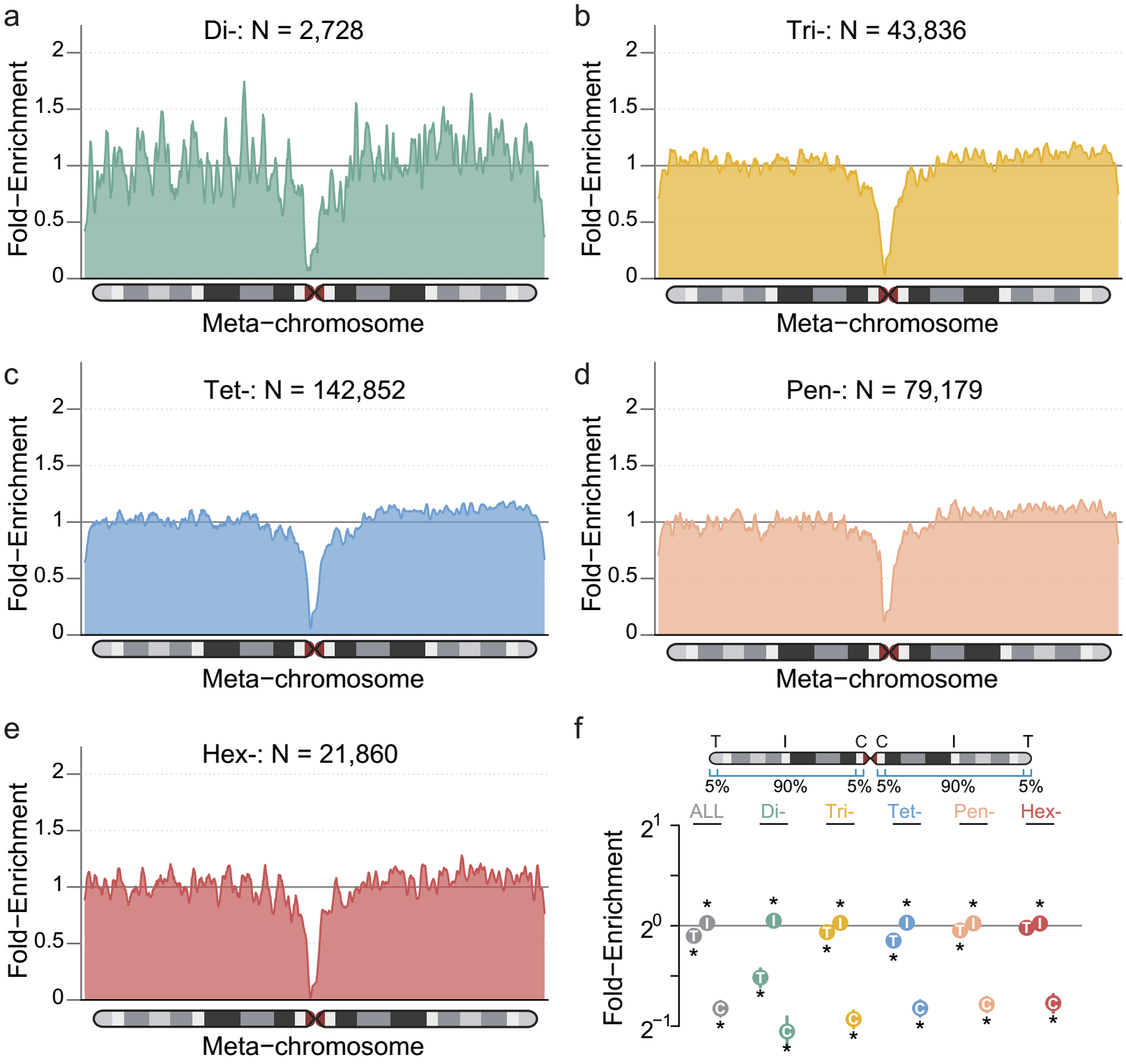


**Fig. S11 Chromosome-level distribution of mSTR density. a-e** Smoothed enrichment of mSTRs with motif lengths of 2 (a), 3 (b), 4 (c), 5 (d) and 6 (e) bp, respectively. The values were calculated per 100 kb window across the average of all autosomes and normalized by the length of chromosome arms (as ‘meta-chromosome’). **f** Enrichment of mSTRs by motif length and chromosomal context. The dots indicate the mean values. P-values were computed using two-sided student’s t-test and adjusted using the Bonferroni method. *, P-value ≤ 0.05. C, centromeric; I, interstitial; T, telomeric.


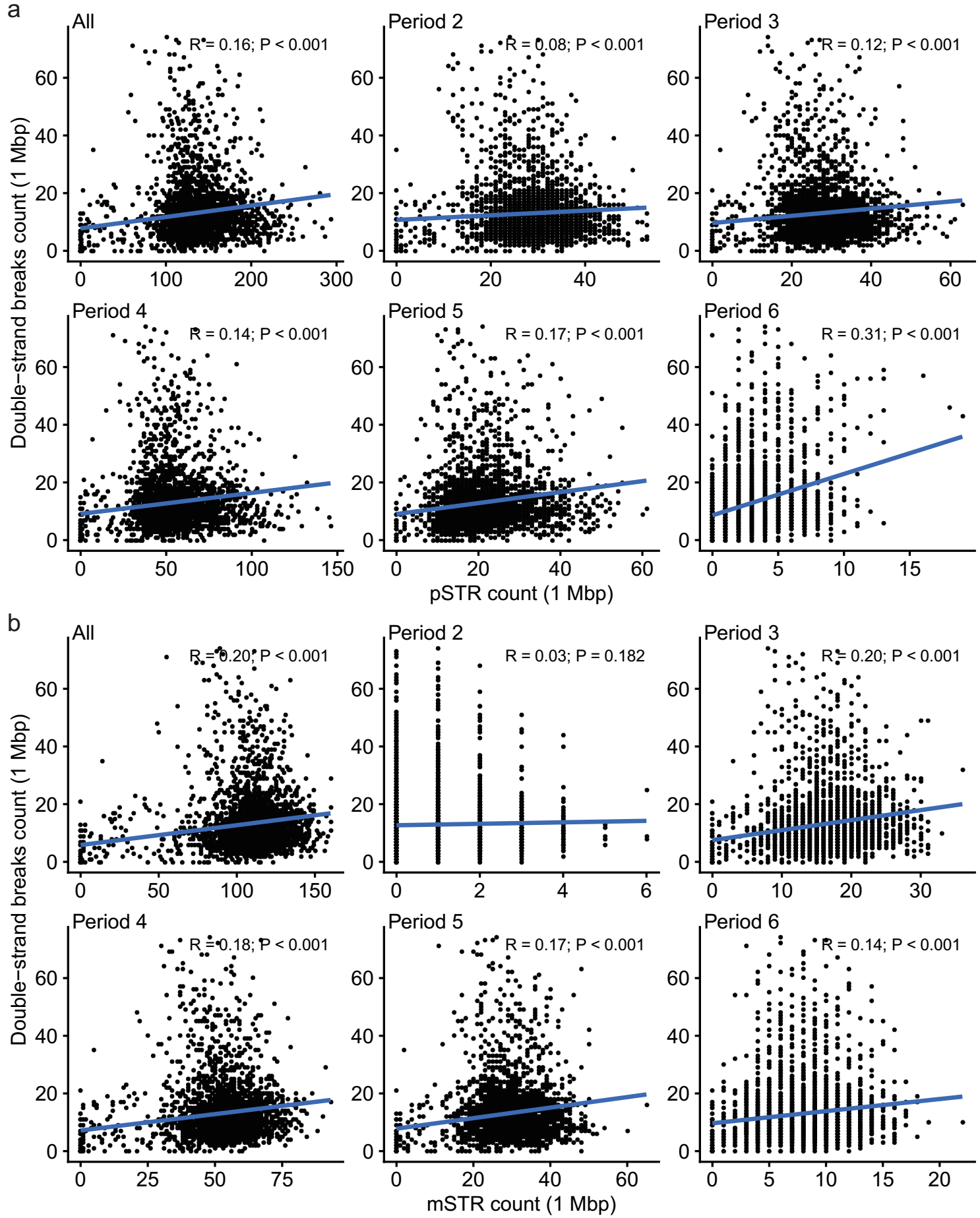


**Fig. S12 Correlation between the number of STR loci and the distribution of double-strand breaks.** Correlation plot between the count of double-strand breaks and pSTRs (**a**) or mSTRs (**b**) in each 1 Mb bin stratified by motif length. Pearson correlation coefficients and the corresponding P-values (two-sided *t*-test) are shown.


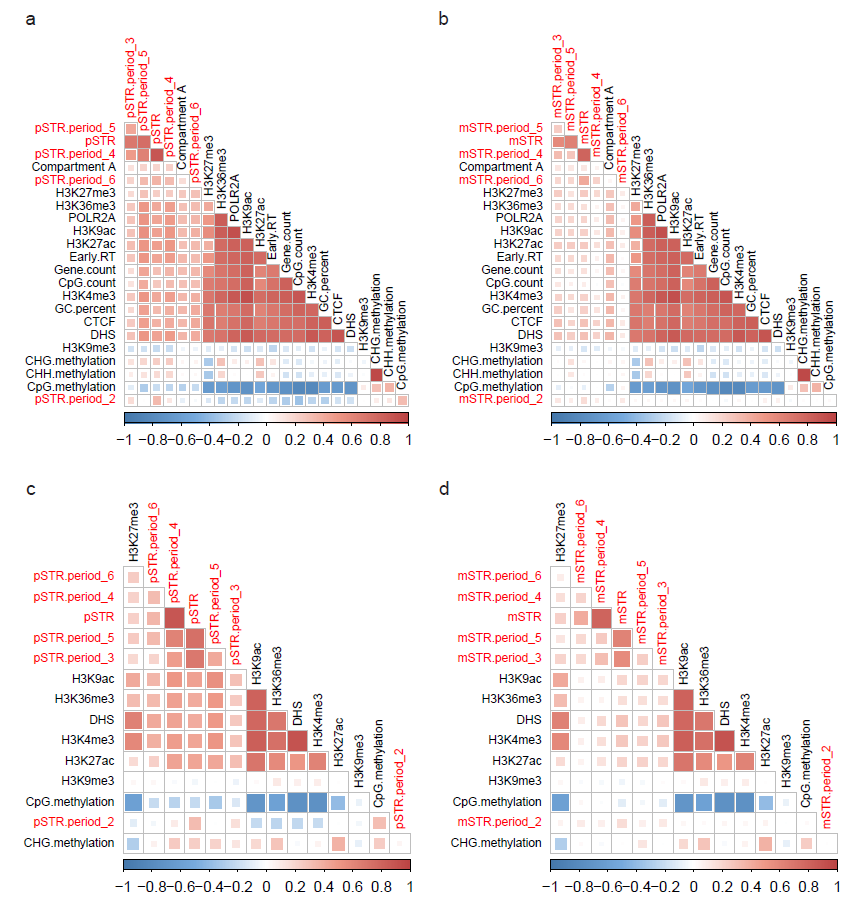


**Fig. S13 Correlations between the occurrence of STRs in our call set and genome features of non-overlapping 1 Mbp windows.** Heatmaps showing the Spearman correlations between pSTRs and genome features of H1-hESC (**a**), mSTRs and genome features of H1-hESC (**b**), pSTRs and genome features of H9-hESC (**c**), and mSTRs and genome features of H9-hESC (**d**), respectively.


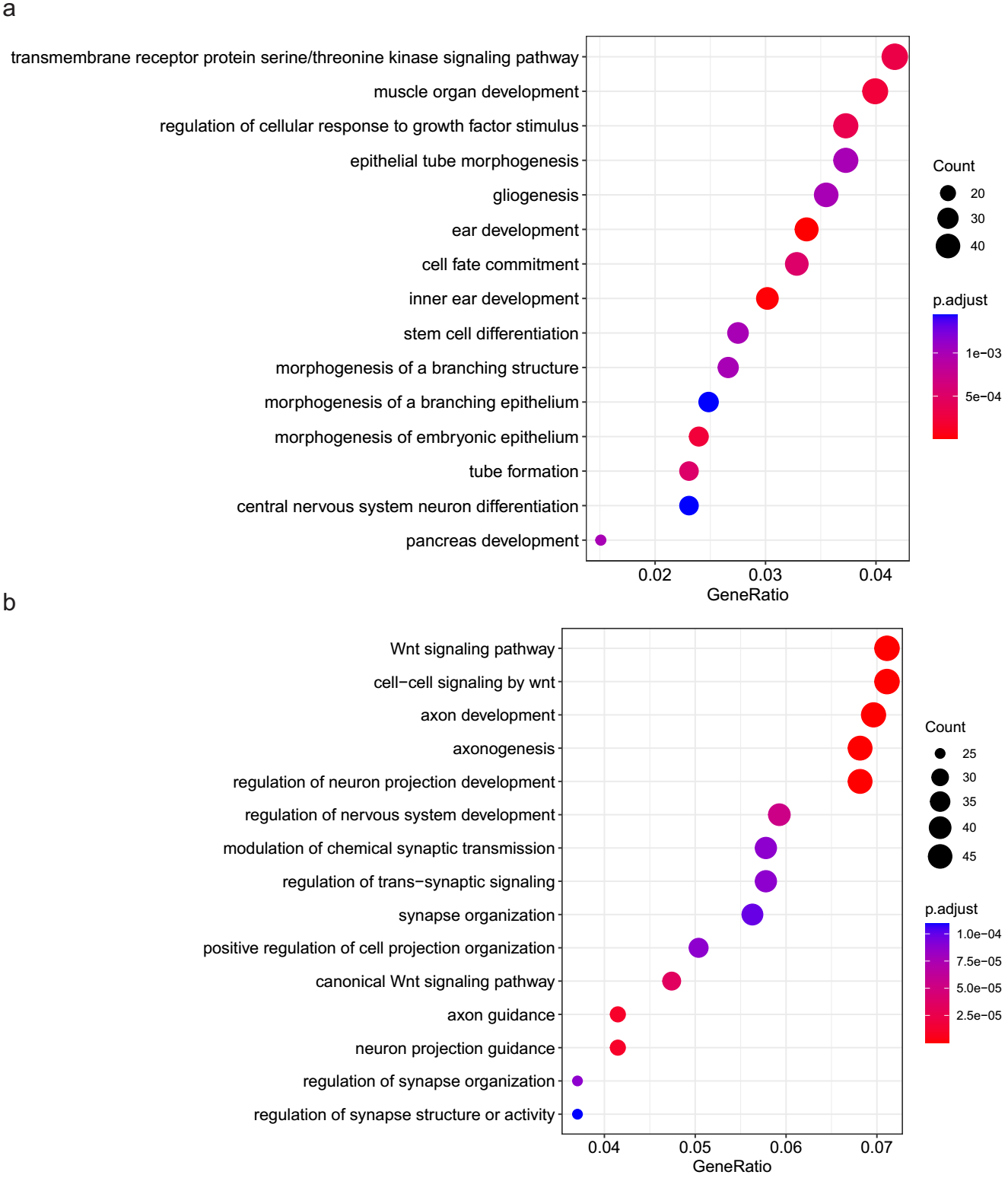


**Fig. S14 Gene Ontology (GO) enrichment analysis for genes containing trimeric or hexameric STRs in their CDS (a) or UTR5 (b).** The top 15 most significant items are shown in each plot. P-values were computed by clusterProfiler v3.18.0 and adjusted using the Benjamini - Hochberg method.


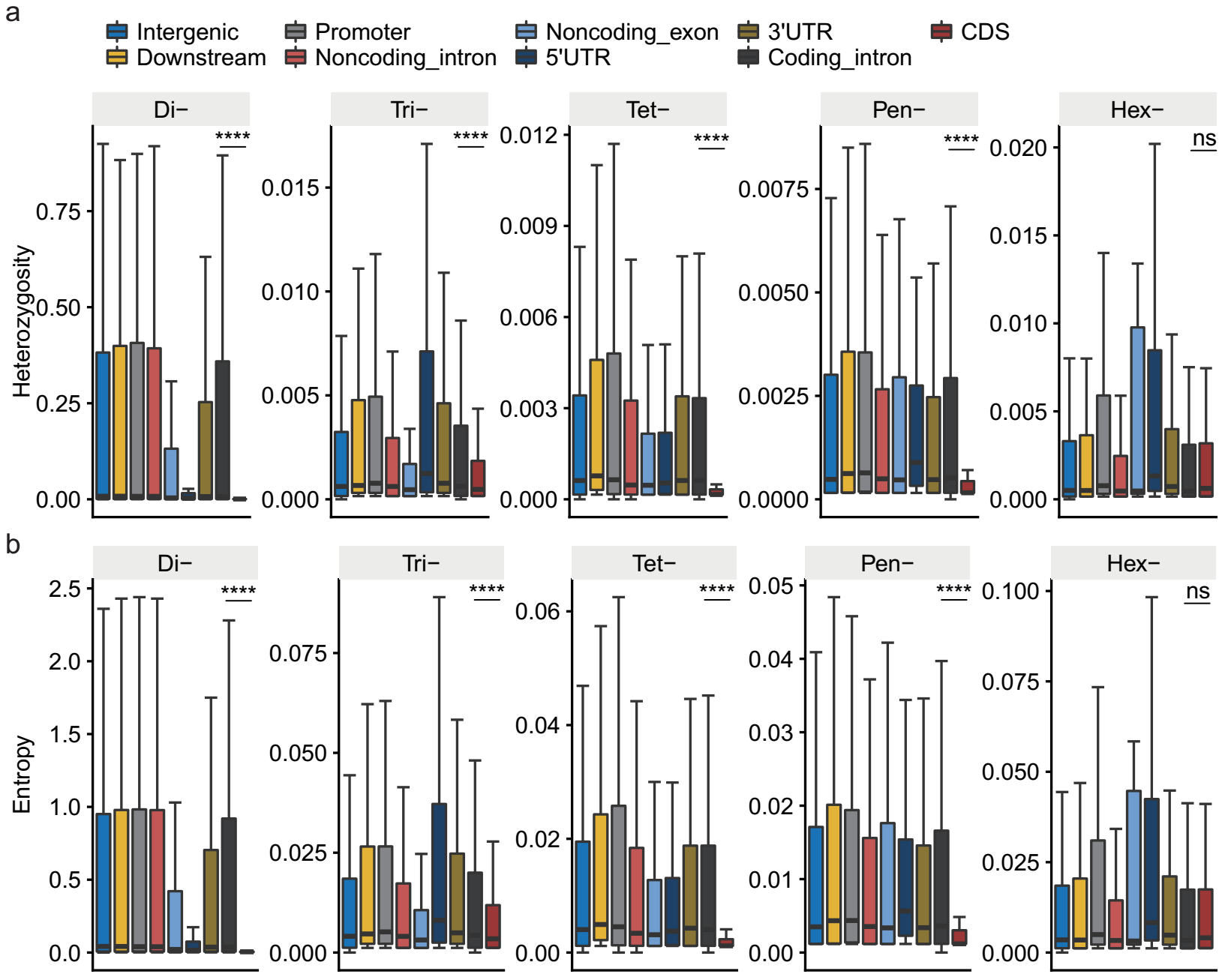


**Fig. S15 Heterozygosity and entropy of pSTRs loci.** Box plots showing heterozygosity (**a**) and entropy (**b**) of pSTRs loci (n = 366,013) with different types of predicted functional consequences, which were stratified by motif length. Horizontal lines indicate the median and boxes span from the lower quartile (the 25th percentiles) to the upper quartile (the 75th percentiles). Whiskers extend to points that are within 1.5 × IQR (interquartile range) from the upper or the lower quartiles. Two-sided Wilcoxon rank sum test was used to compute P-values. ns, P-value ≥ 0.05; ****, P-value < 0.0001.


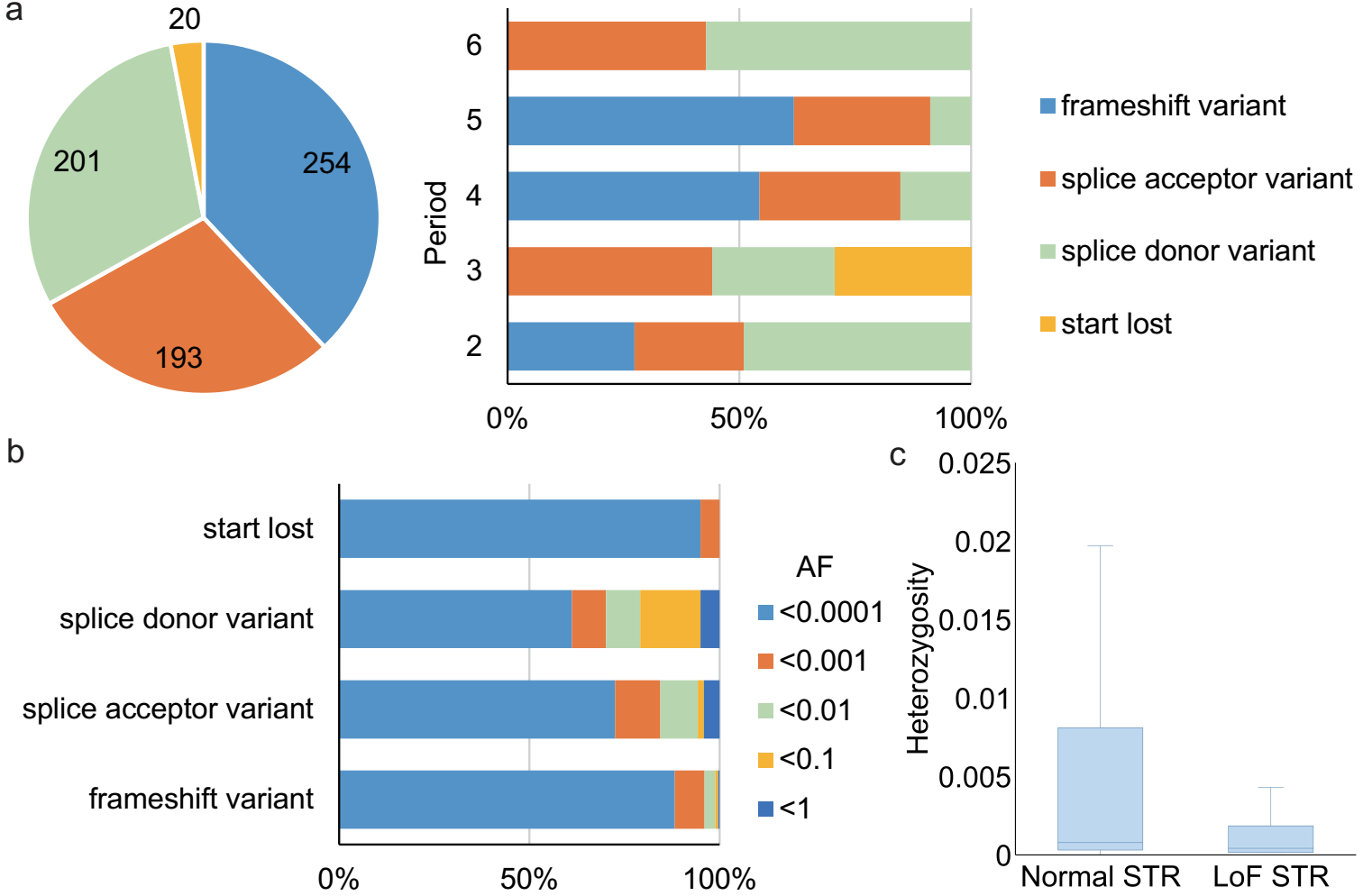


**Fig. S16 Loss of function (LoF) STR alleles. a** The number of LoF STR alleles of different types in total (left) or stratified by motif length (right). **b** Proportion of LoF alleles in different allele frequency bins. Allele frequency bins: <0.0001, 0-0.0001; <0.001, 0.0001-0.001; <0.01, 0.001-0.01; <0.1, 0.01-0.1; <1, 0.1-1. **c** Box plot showing the distribution of heterozygosity of LoF pSTR loci (n = 392) and normal pSTR (n = 365,621) loci. Horizontal lines indicate the median and boxes span from the lower quartile (the 25th percentiles) to the upper quartile (the 75th percentiles). Whiskers extend to points that are within 1.5 × IQR (interquartile range) from the upper or the lower quartiles.


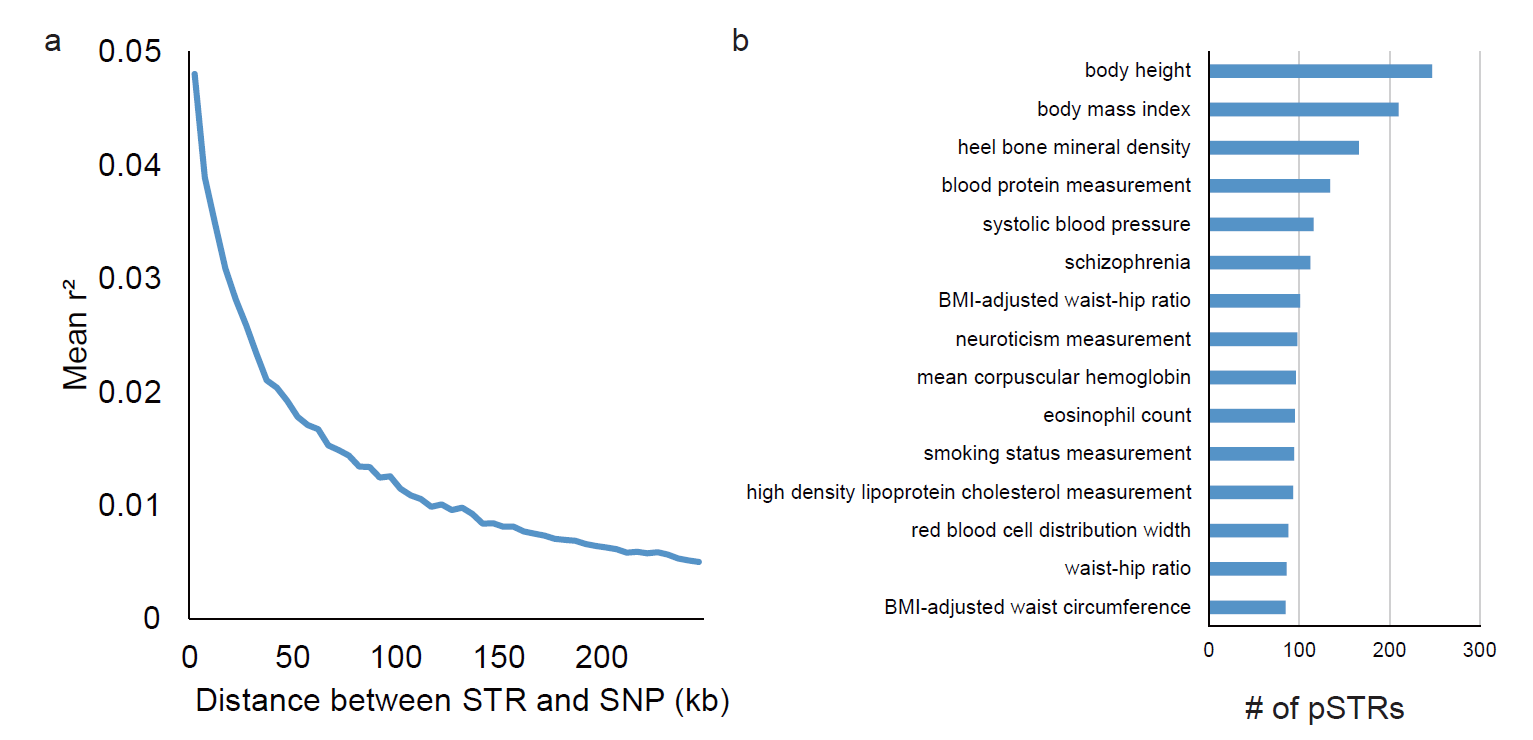


**Fig. S17 Linkage disequilibrium between pSTRs and GWAS risk SNPs. a** Patterns of linkage disequilibrium for pSTRs and GWAS risk SNPs. **b** Number of pSTRs that were in high linkage disequilibrium between GWAS SNPs of the corresponding traits. The top 15 traits with the most pSTRs are shown.


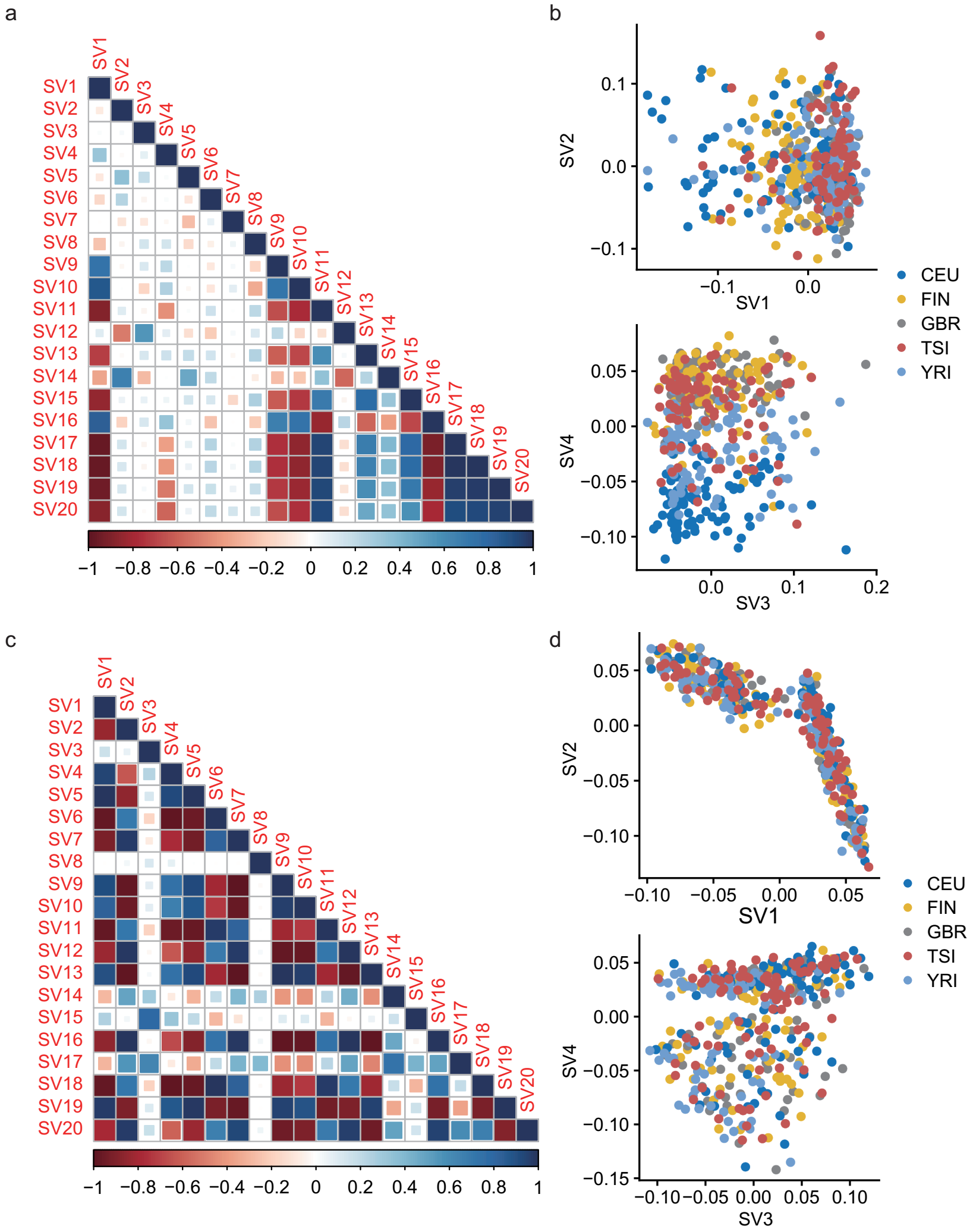


**Fig. S18 Detecting hidden covariates with IA-SVA for gene expression and PDUI values. a** Correlations of the top 20 hidden factors for gene expression data. **b** First and second hidden factors (upper), and third and fourth hidden factors (lower) for gene expression data. **c** Correlations of the top 20 hidden factors for PDUI data. **d** First and second hidden factors (upper), and third and fourth hidden factors (lower) for PDUI data.


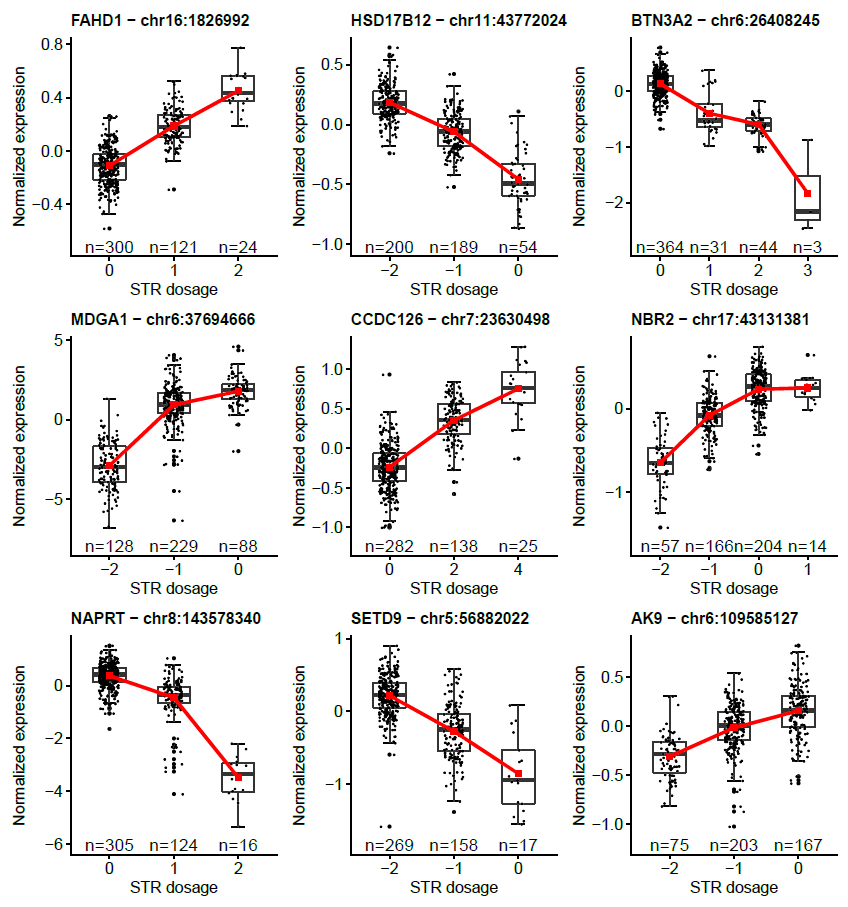


**Fig. S19 Examples of multi-allelic eSTRs.** For each plot, the x-axis represents the STR dosage relative to the homozygous reference genotype, and the y-axis represents normalized expression of the gene regulated by the corresponding eSTR. Horizontal lines indicate the median and boxes span from the lower quartile (the 25th percentiles) to the upper quartile (the 75th percentiles). Whiskers extend to points that are within 1.5 × IQR (interquartile range) from the upper or the lower quartiles. Each dot represents an individual. N indicates the number of samples with the corresponding dosage at the STR locus. The position of the eSTR and the symbol of gene regulated by the eSTR are marked in each plot. The red line shows the mean expression value.


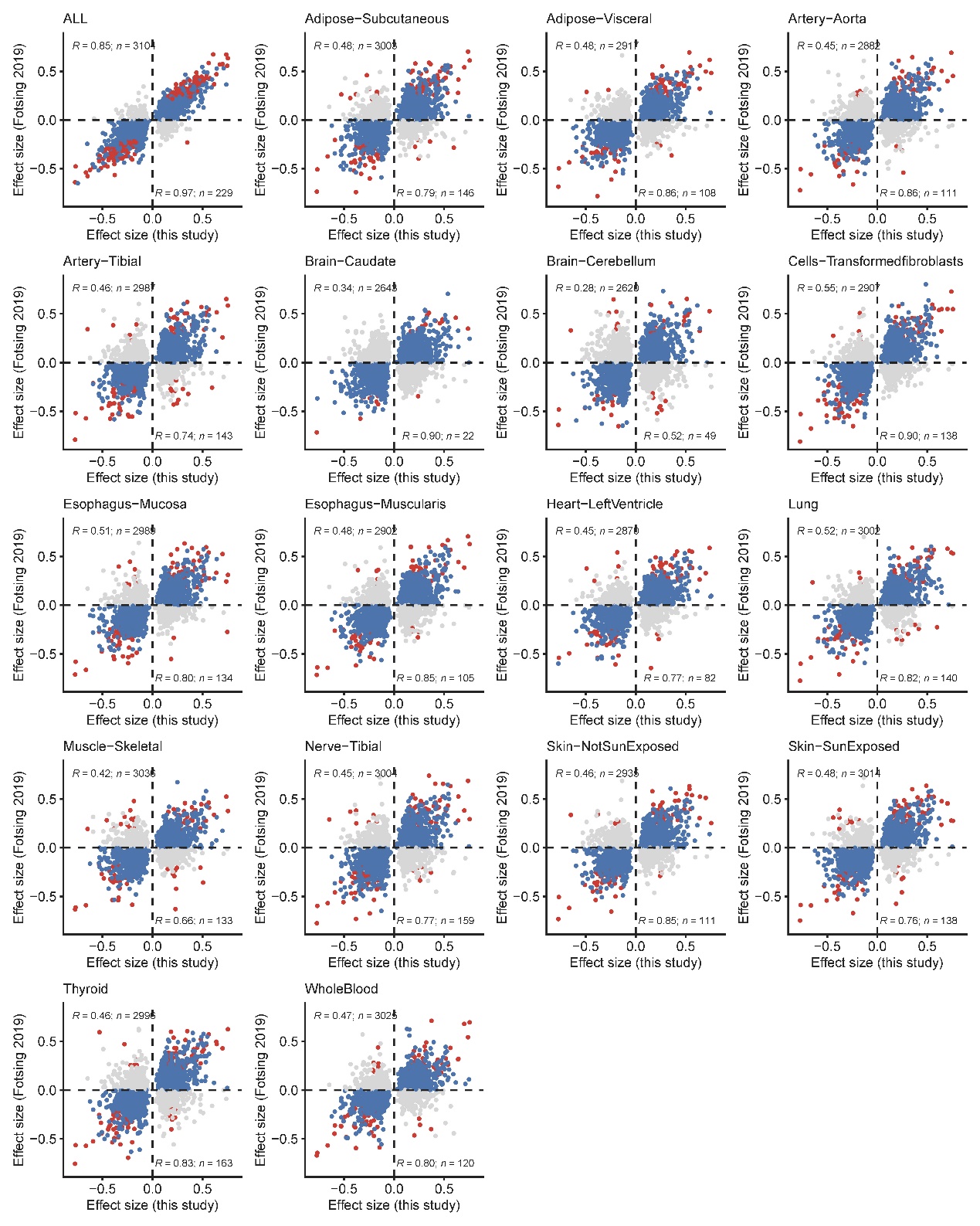


**Fig. S20 Effect size of eSTRs identified in this study and a study by Fotsing et al.** Correlations of the effect size of eSTRs identified in this study and a previous study by Fotsing et al. in GTEx dataset. Each subplot represents a tissue in GTEx. The blue points indicate eSTRs whose directions of effect were concordant in two studies, and the gray points denote eSTRs with discordant directions of effect for that eSTR. The eSTRs detected in both studies are colored red, regardless of the concordance of effect.


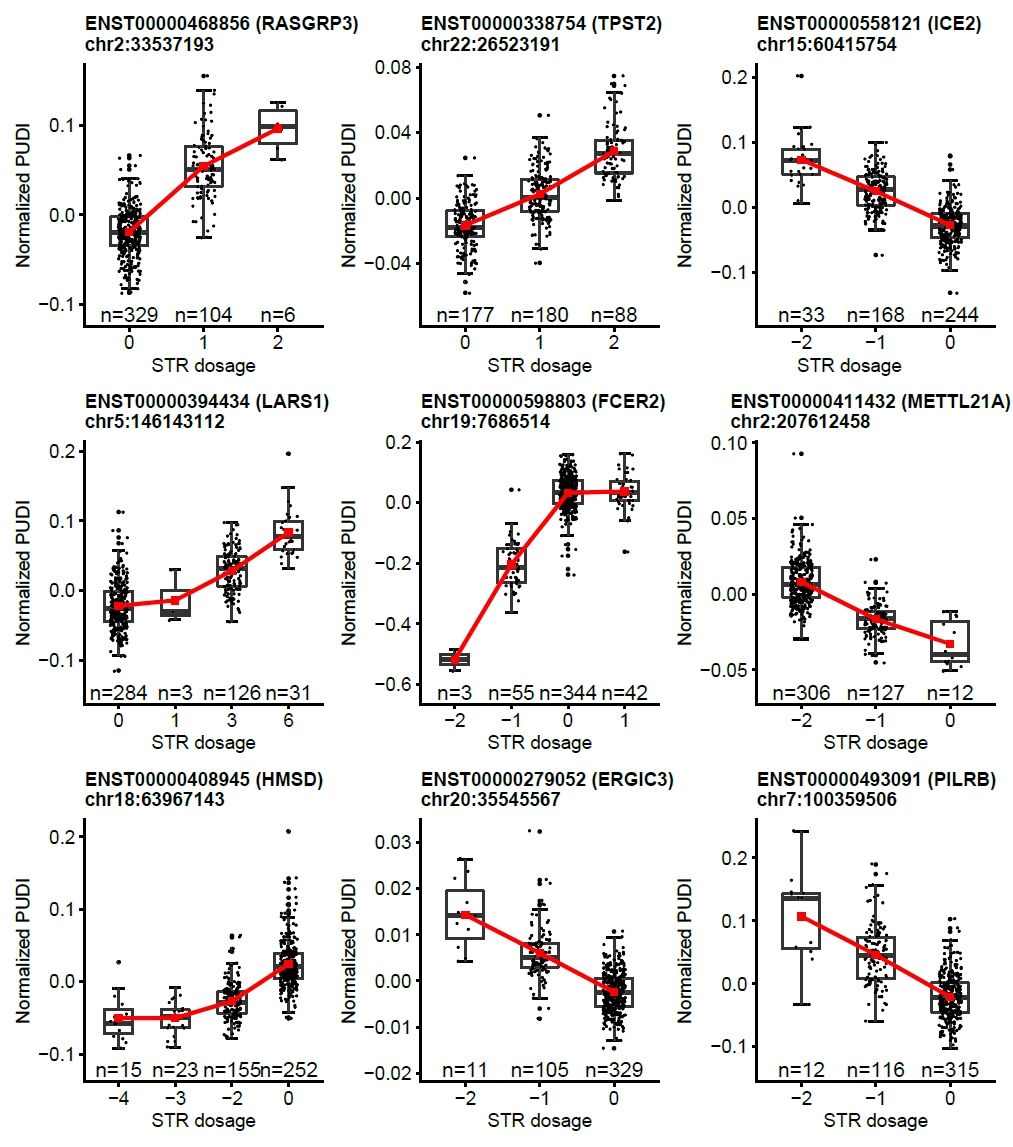


**Fig. S21 Examples of multi-allelic 3’aSTRs.** For each plot, the x-axis represents the STR dosage relative to the homozygous reference genotype, and the y-axis represents normalized PDUI value of the transcript regulated by the corresponding 3’aSTR. Horizontal lines indicate the median and boxes span from the lower quartile (the 25th percentiles) to the upper quartile (the 75th percentiles). Whiskers extend to points that are within 1.5 × IQR (interquartile range) from the upper or the lower quartiles. Each dot represents an individual. “n” indicates the number of samples with the corresponding dosage at the STR locus. The position of the 3’aSTR and the symbol of transcript regulated by the 3’aSTR are marked in each plot. The red line shows the mean PDUI value.


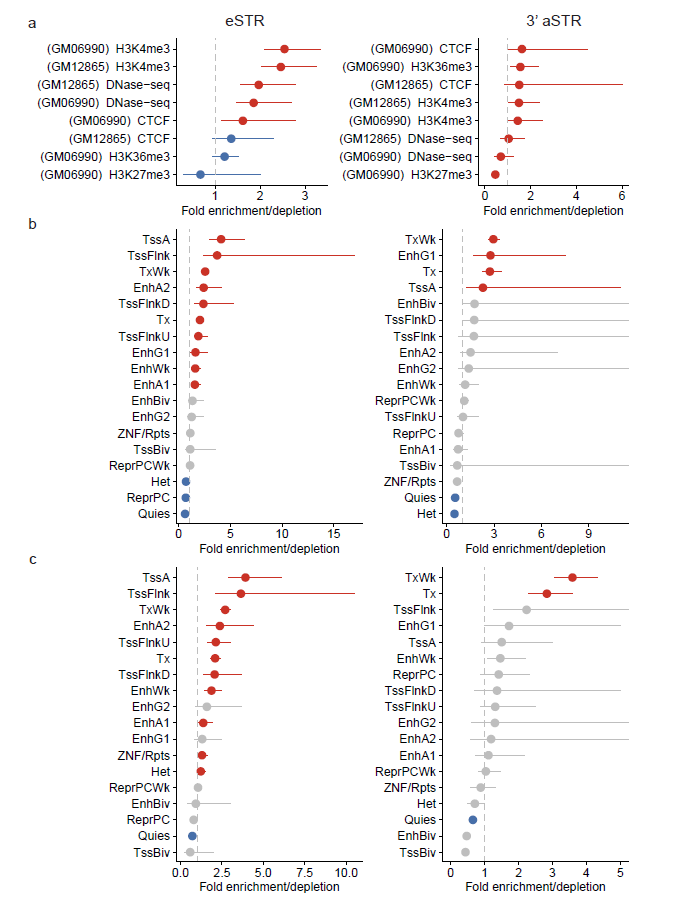


**Fig. S22 Correlation of eSTRs or 3’aSTRs with multiple genomic contexts.** Fold enrichment of eSTRs (left) or 3’aSTRs (right) in histone mark peaks (**a**) and chromatin states defined by ChromHMM from GM06990 cell line (**b**) and GM12865 cell line (**c**). For each Figure, a permutation test was repeated 1,000 times, and empirical P values were computed together with the enrichment values by GAT v1.3.4. Points denote the enrichment values. Red and blue points denote significant enrichments or depletions, respectively (P < 0.05 after Benjamini & Hochberg correction), and bars show 95% confidence intervals.


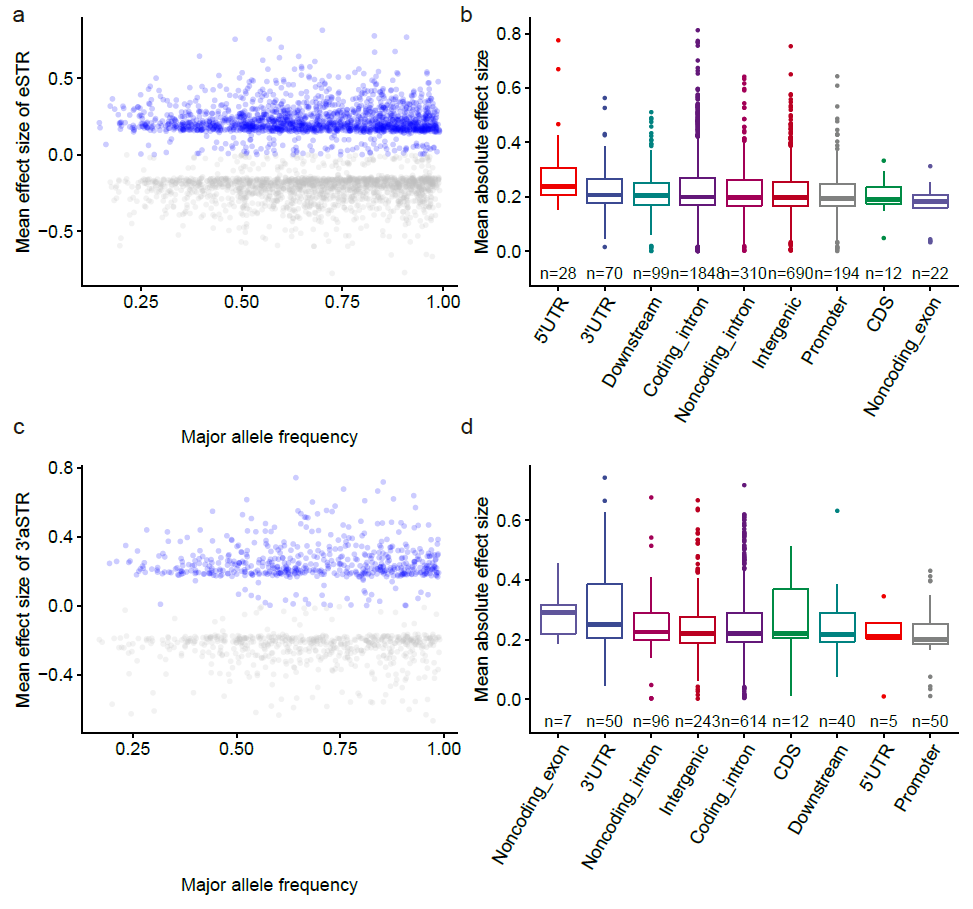


**Fig. S23 Effect sizes of eSTRs and 3’aSTRs. a** Distribution of major allele frequencies and effect sizes of eSTRs. **b** Mean absolute effect sizes of eSTRs within different gene features. The number of eSTR loci in each group is shown. **c** Distribution of major allele frequencies and effect sizes of 3’aSTRs. **d** Mean absolute effect sizes of 3’aSTRs within different gene features. The number of 3’aSTR loci in each group is shown. For Figure b and d, horizontal lines indicate the median, boxes span from the lower quartile (the 25th percentiles) to the upper quartile (the 75th percentiles), and whiskers extend to points that are within 1.5 × IQR (interquartile range) from the upper or the lower quartiles.


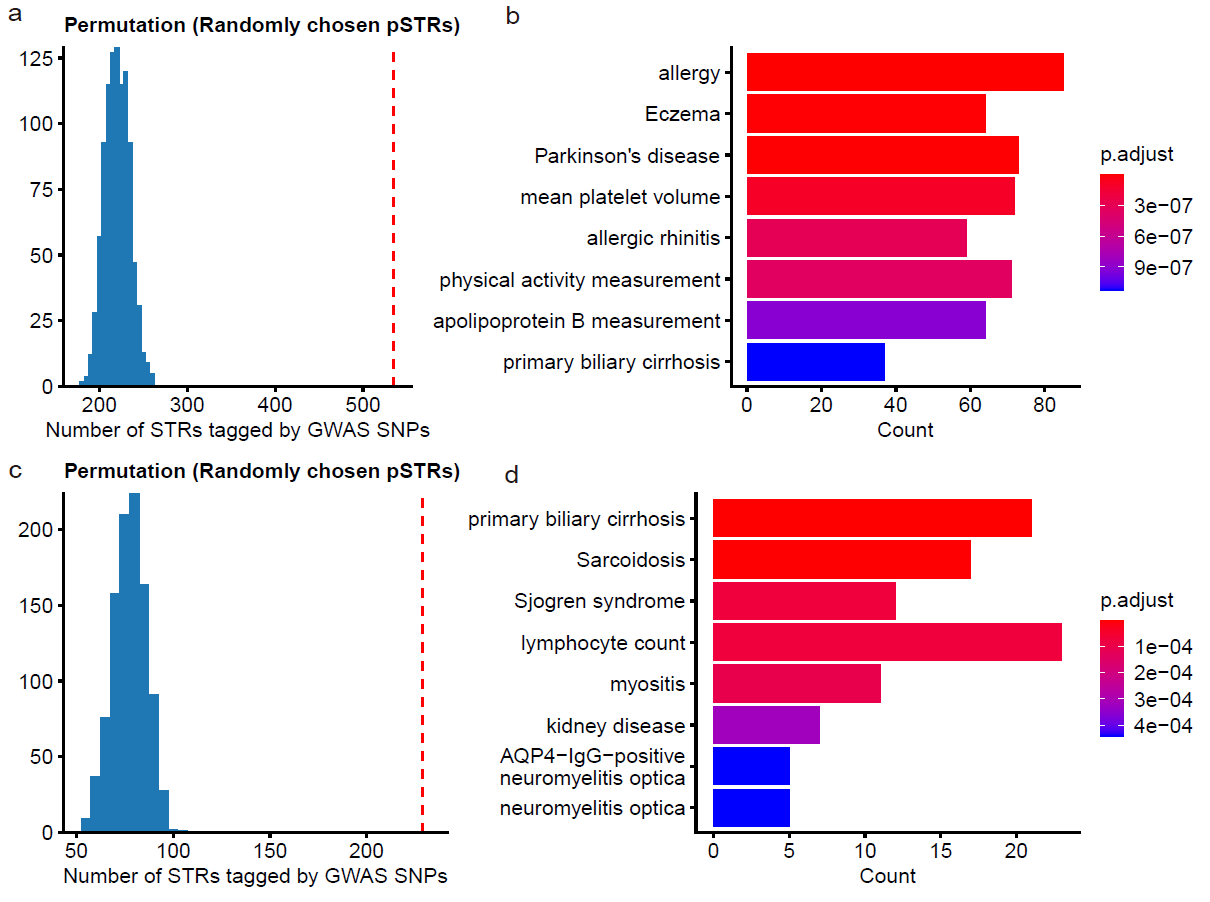


**Fig. S24 GWAS hit enrichment analysis of eSTRs and 3’aSTRs. a** The number of pSTRs in eSTRs in high LD with SNPs in the GWAS catalog. Histogram shows the number of pSTRs that were used in this analysis and tagged by GWAS SNPs resulting from 1000 permutations. The red dashed line shows the actual number of eSTRs tagged by GWAS catalog SNPs. **b** Enrichment of genes targeted by eSTRs in GWAS genes related to a variety of traits. **c** The number of pSTRs in 3’aSTRs in high LD with variants in the GWAS catalog. Histogram shows the number of pSTRs that were used in this analysis and tagged by GWAS SNPs resulting from 1000 permutations. The red dashed line shows the actual number of 3’aSTRs tagged by GWAS catalog SNPs. **d** Enrichment of genes targeted by 3’aSTRs in GWAS genes related to a variety of traits. For both Figure b and d, P-values were computed by clusterProfiler v3.18.0 and adjusted using the Benjamini - Hochberg method.


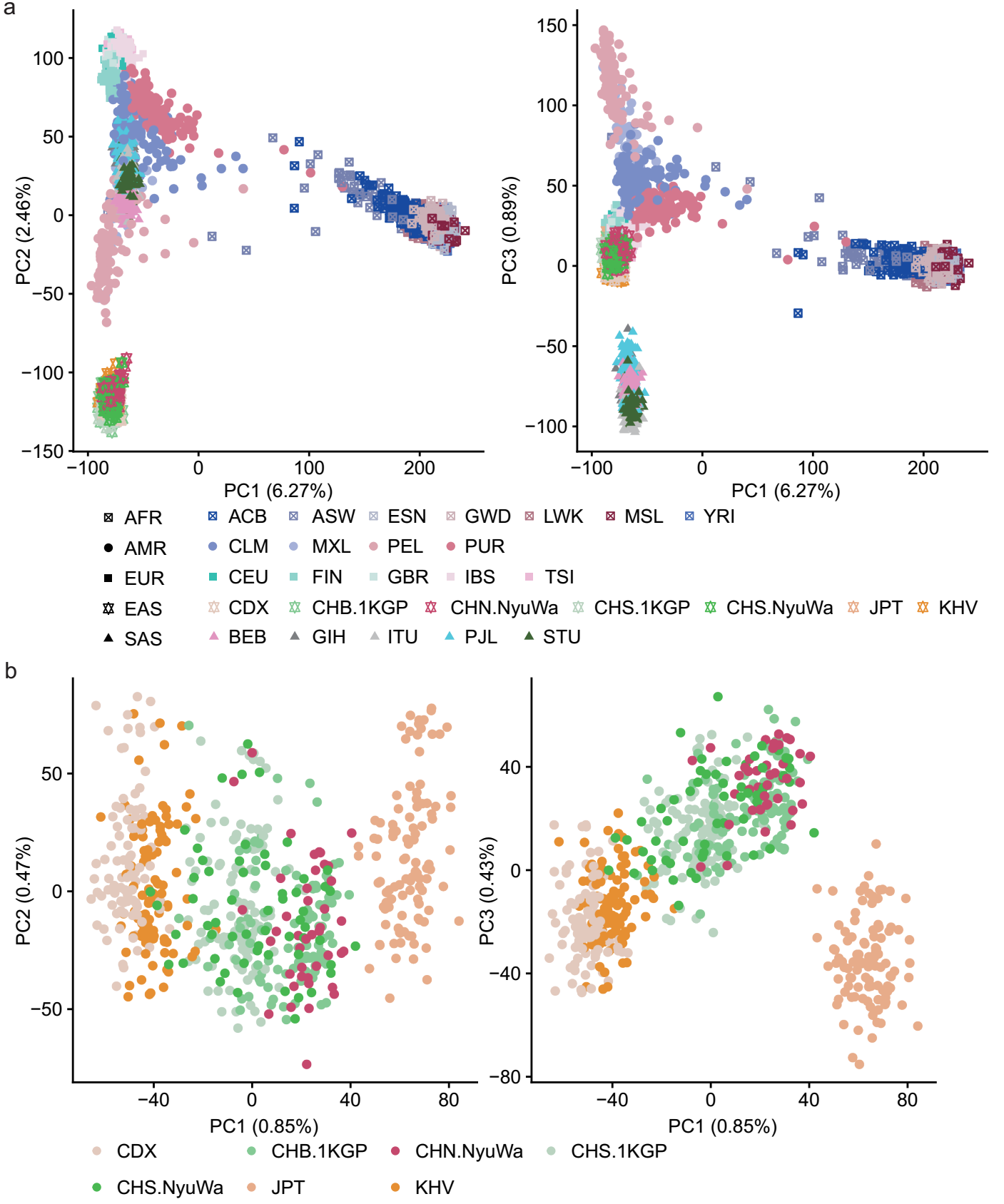


**Fig. S25 Principal component analysis of pSTRs. a** PCA of all 1KGP samples with 200 randomly selected NyuWa samples. Abbreviations of populations are from 1KGP (Supplementary Data 1). Shapes represent the superpopulation of each sample. Colors represent the population of each sample. **b** PCA of all 1KGP EAS samples with 200 randomly selected NyuWa samples. Abbreviations of populations are from 1KGP (Supplementary Data 1). Colors represent the population of each sample.


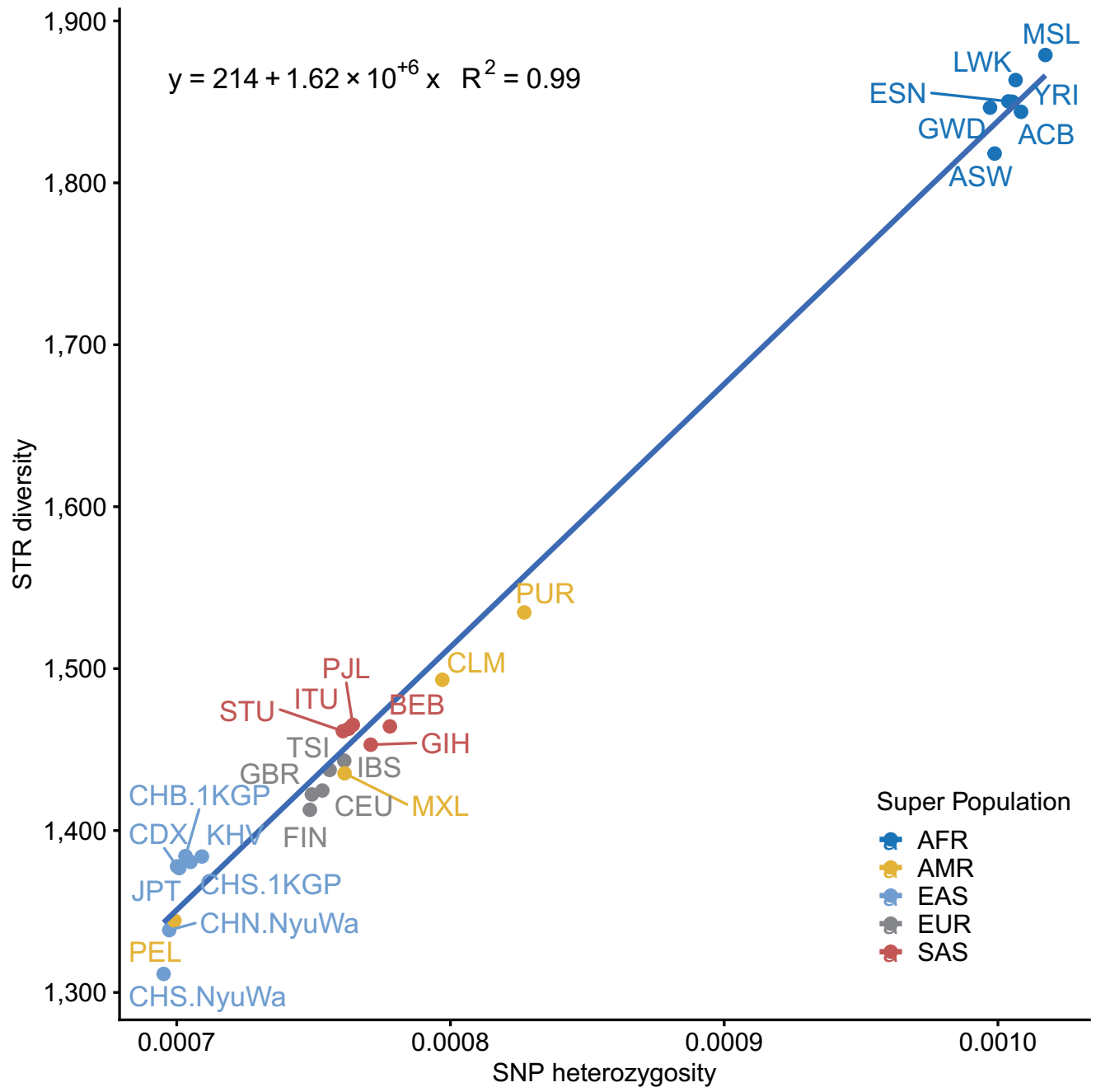


**Fig. S26 Correlation between SNP heterozygosity and STR diversity.** SNP heterozygosity was defined as the ratio of heterozygous SNPs across the individual’s genome and STR diversity was computed as the average number of STR differences between every two individuals in a given population. Points are colored by superpopulations. AFR, African superpopulation; AMR, American superpopulation; EAS, East Asian superpopulation; EUR, European superpopulation; SAS, South Asian superpopulation.


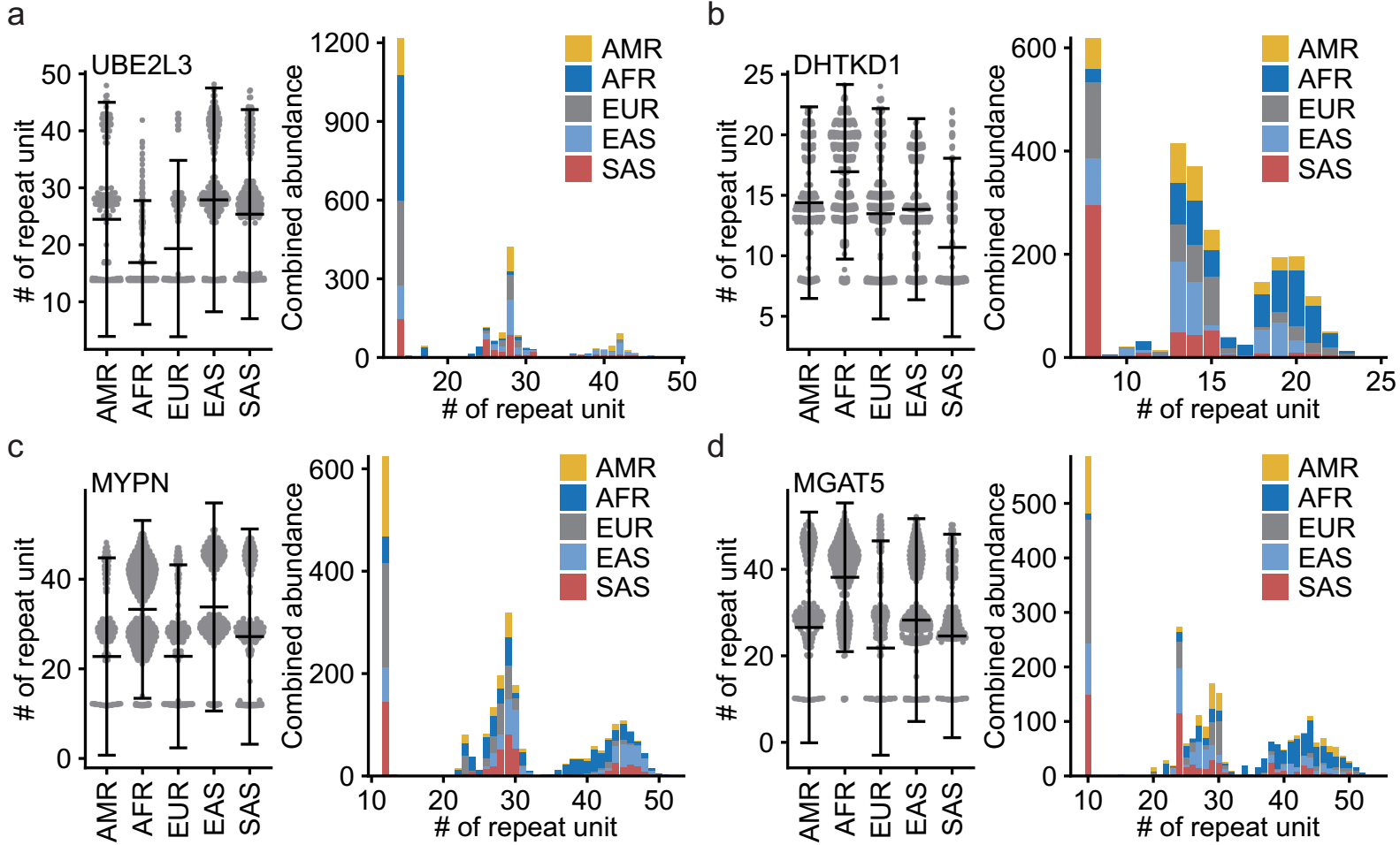


**Fig. S27 Examples of pSTRs with greatest length differences across superpopulations in the 1KGP. a-d** Repeat numbers together with mean and standard deviation of pSTR from different superpopulations (left) and cumulative abundance of pSTRs with different repeat numbers (right) in *UBE2L3* (a), *DHTKD1* (b), *MYPN* (c) and *MGAT5* (d).


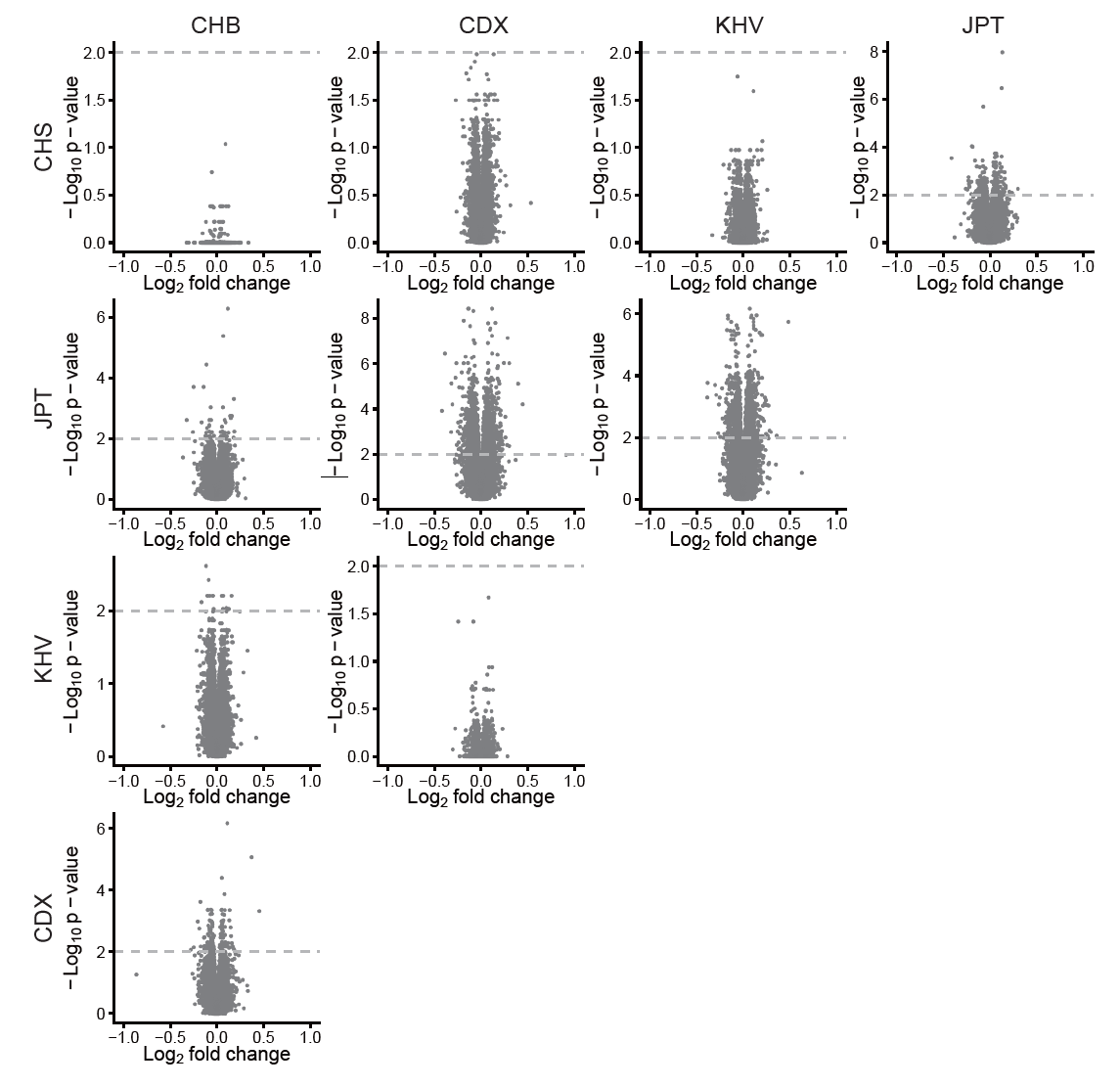


**Fig. S28 Comparing STR lengths across populations in East Asia.** Each dot indicates a pSTR locus. For each subplot, the x-axis and y-axis reflect the log2 fold change of repeat length and the P-value from Wilcoxon rank sum test between the two populations, respectively. The sample size of each population is 93-105. CHB, Han Chinese in Beijing, China; CHS, Southern Han Chinese; CDX, Chinese Dai in Xishuangbanna; KHV, Kinh in Ho Chi Minh City, Vietnam; JPT, Japanese in Tokyo, Japan.


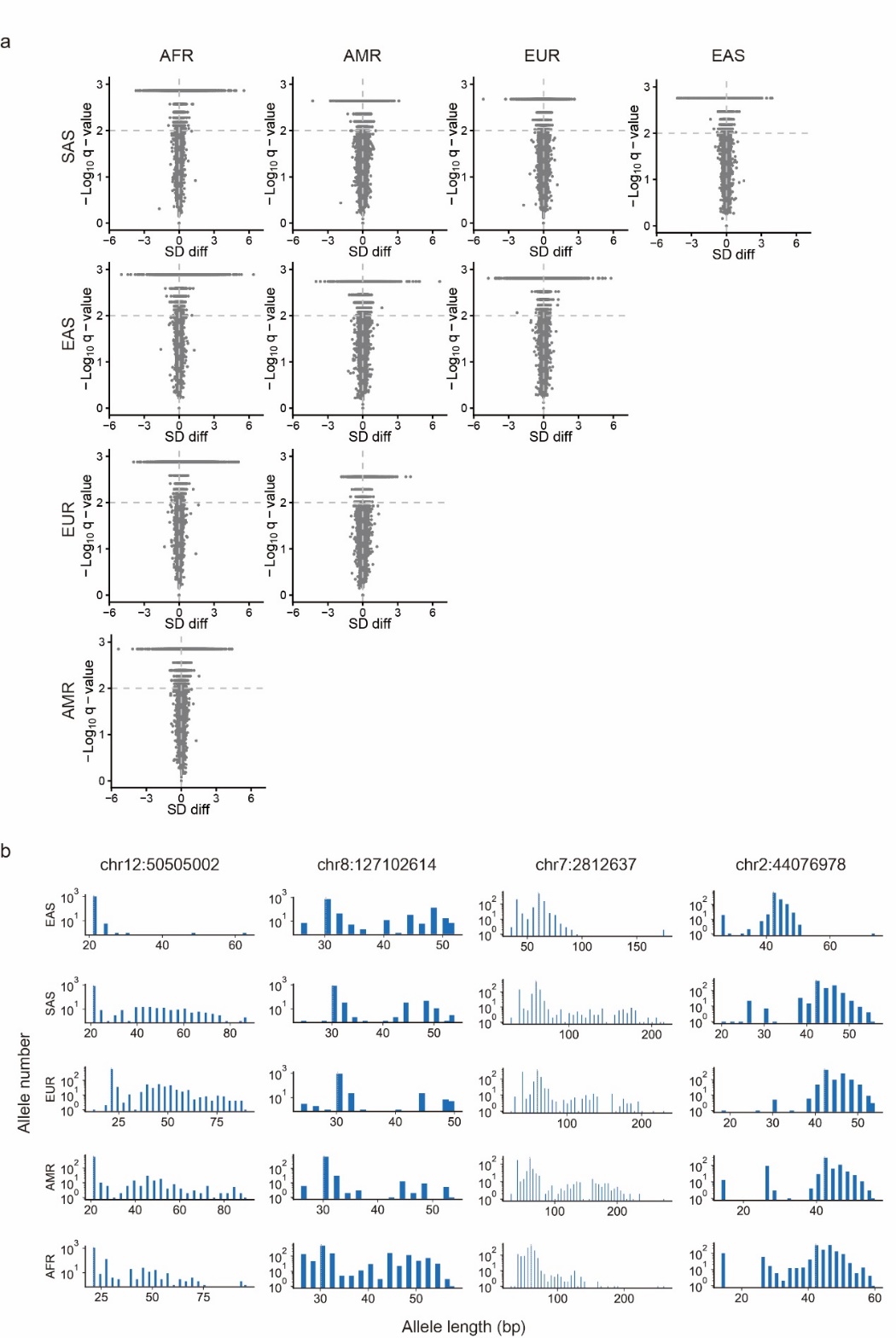


**Fig. S29 Comparing STR length variance across the five superpopulations in the 1KGP dataset. a** Pairwise comparisons of pSTR length variance between superpopulations from the 1KGP are shown using volcano plots. The gray horizontal lines indicate the q-value cutoff -log_10_(0.01) for pSTRs with differential length variances. **b** Distribution of allele length for four examples of pSTRs with significant changes in variance of allele lengths. The gray vertical line indicates the reference allele length for the corresponding locus.


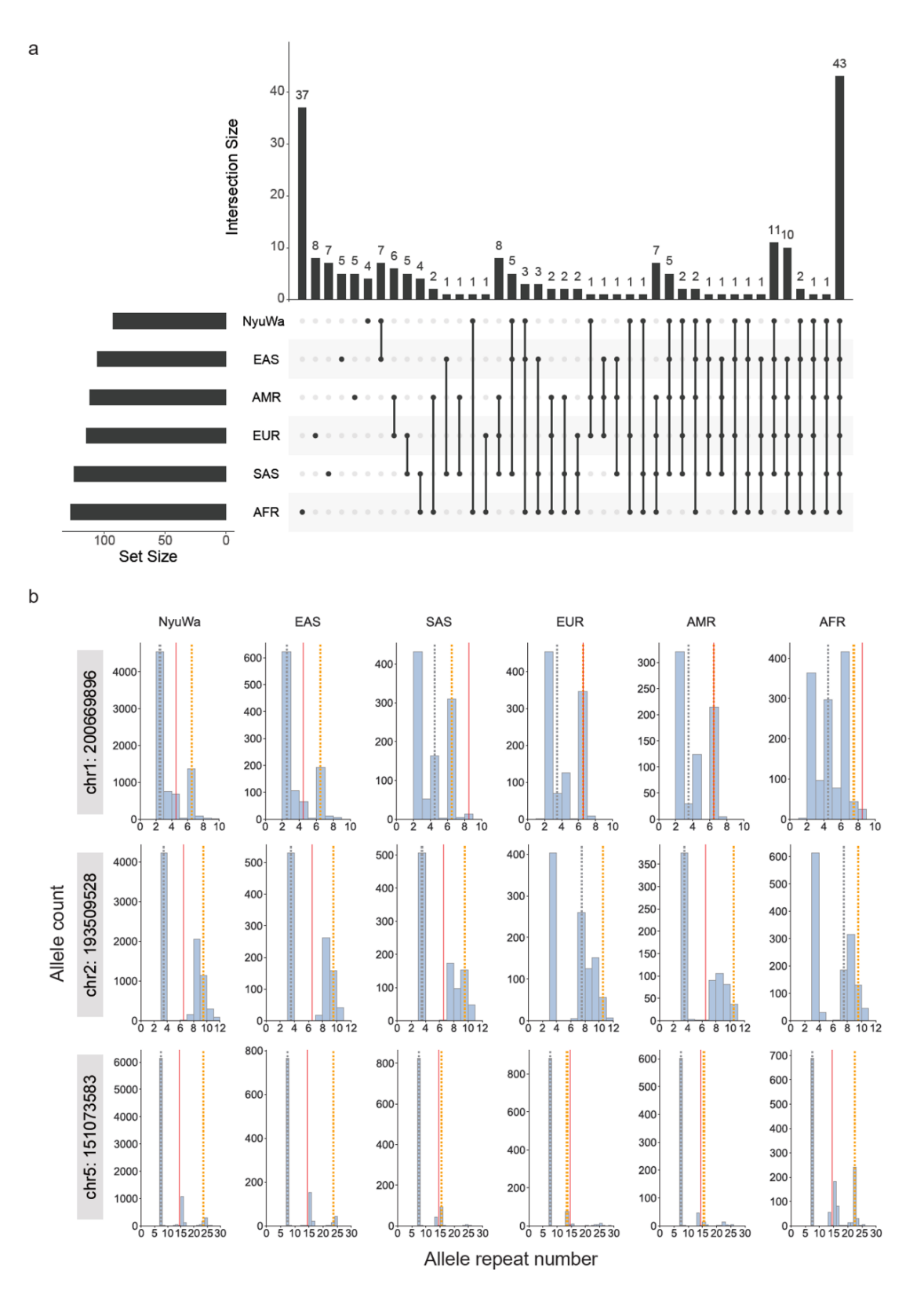


**Fig. S30 Analysis of pSTR expansion. a** UpSet plot of expanded pSTRs identified from the NyuWa dataset and the five superpopulations in the 1KGP. **b** Distribution of allele repeat number for three examples of expanded pSTRs stratified by superpopulation. The gray vertical line, red vertical line, and yellow vertical line indicate the median of allele repeat number, double of the median of allele repeat number, and the 95% quantile of allele repeat number for the corresponding locus, respectively.


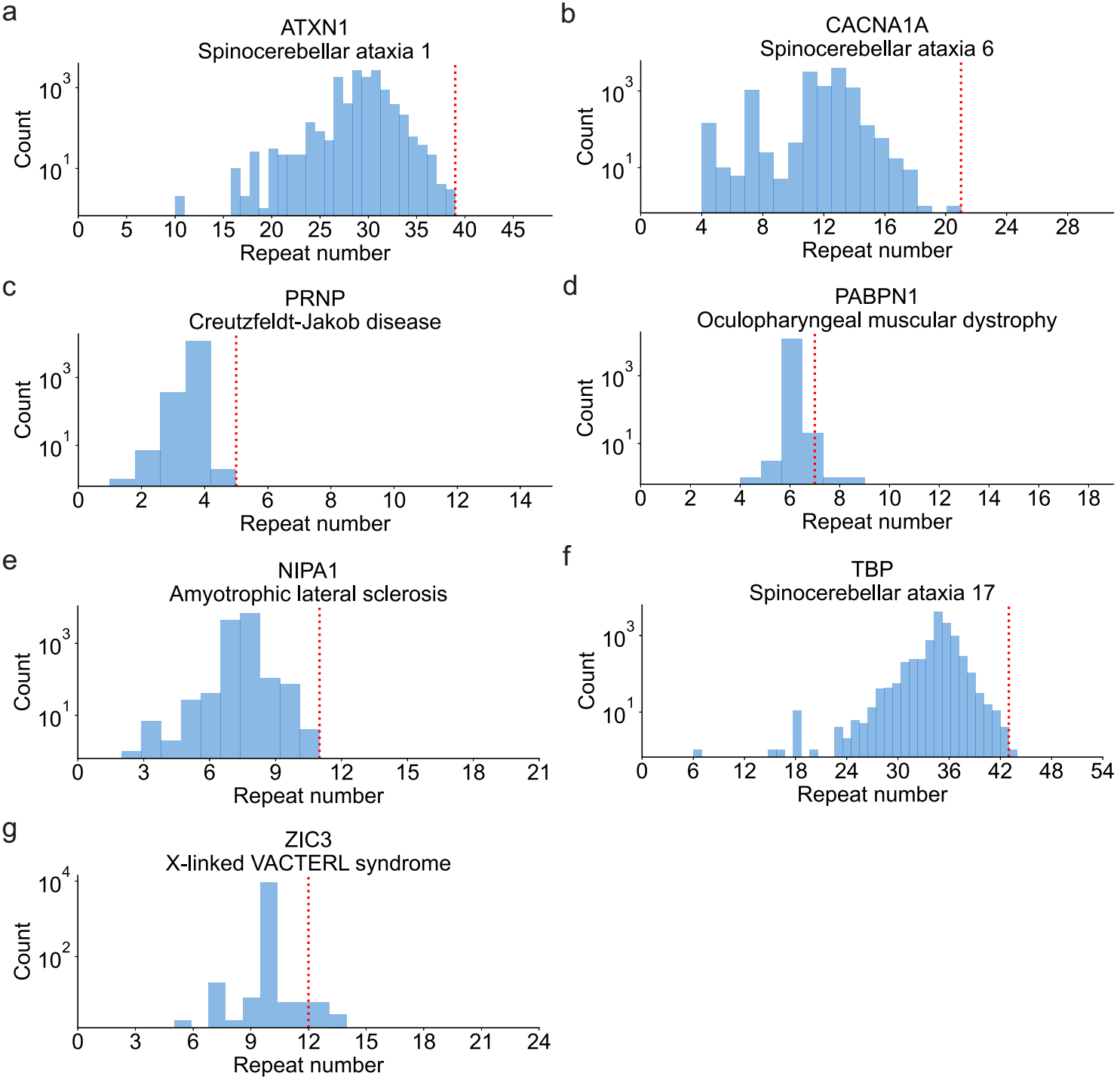


**Fig. 31 Repeat number distribution of pathogenic STRs.** Repeat number distributions of loci for which repeats longer than the pathogenic threshold were detected in our dataset. The red vertical lines indicate the pathogenic threshold of repeat number for the corresponding locus.


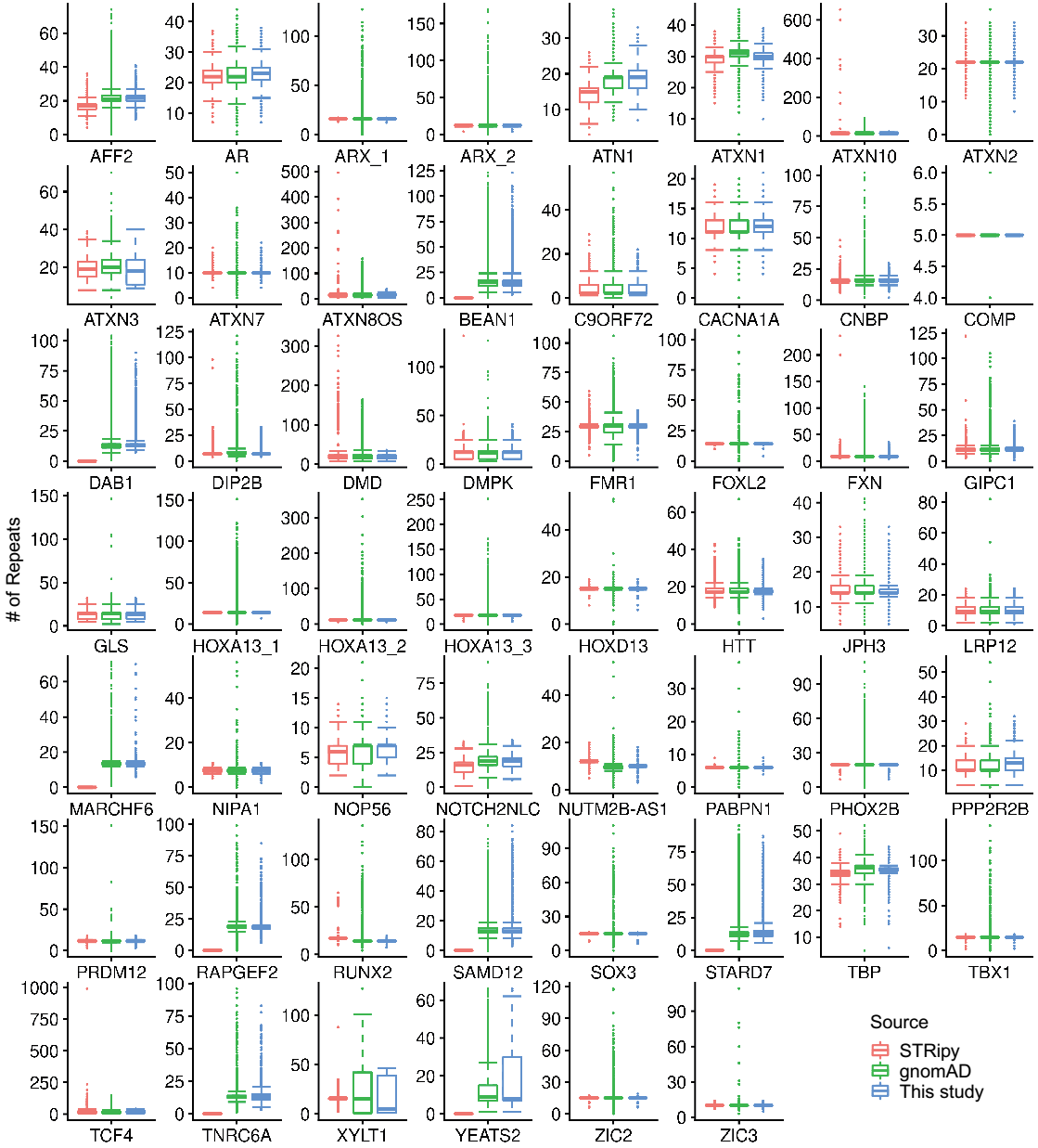


**Fig. S32 Comparison of the repeat number distribution at known pathogenic STR loci.** Repeat number distribution at pathogenic STRs in this study (blue), STRipy (red) and gnomAD (green). Horizontal lines indicate the median and boxes span from the lower quartile (the 25th percentiles) to the upper quartile (the 75th percentiles). Whiskers extend to points that are within 1.5 × IQR (interquartile range) from the upper or the lower quartiles.
