# Supplementary material for: Characterization of genome-wide STR variation in 6487 human genomes
Source: Nat Commun. 2023 Apr 12;14:2092. doi: 10.1038/s41467-023-37690-8 (PMC10097659; doi:10.1038/s41467-023-37690-8)
Supplement: Supplementary file 3 — Description of Additional Supplementary Files [file 41467_2023_37690_MOESM3_ESM.pdf]

Description of Additional Supplementary Files

File Name: Supplementary Data 1
Description: Information of samples used in this study.

File Name: Supplementary Data 2
Description: Known disease loci for ExpansionHunter.

File Name: Supplementary Data 3
Description: Concordance between ExpansionHunter and GangSTR at pathogenic STRs.

File Name: Supplementary Data 4
Description: The sources and accession numbers used for correlation of STR occurrences and genome features.

File Name: Supplementary Data 5
Description: LoF pSTR alleles.

File Name: Supplementary Data 6
Description: LD with GWAS risk SNPs.

File Name: Supplementary Data 7
Description: eSTR identified in this study.

File Name: Supplementary Data 8
Description: 3'aSTR identified in this study.

File Name: Supplementary Data 9
Description: Rst levels of pSTRs between different populations.

File Name: Supplementary Data 10
Description: Highly variable pSTRs within superpopulation.

File Name: Supplementary Data 11
Description: STR expansion analysis
