# Supplementary material for: Characterization of genome-wide STR variation in 6487 human genomes
Source: Nat Commun. 2023 Apr 12;14:2092. doi: 10.1038/s41467-023-37690-8 (PMC10097659; doi:10.1038/s41467-023-37690-8)
Supplement: Supplementary file 5 — Reporting Summary [file 41467_2023_37690_MOESM5_ESM.pdf]

nature portfolio

Corresponding author(s): Shunmin He

Last updated by author(s): Mar 23, 2023

Reporting Summary

Nature Portfolio wishes to improve the reproducibility of the work that we publish. This form provides structure for consistency and transparency in reporting. For further information on Nature Portfolio policies, see our Editorial Policies and the Editorial Policy Checklist.

Statistics

For all statistical analyses, confirm that the following items are present in the figure legend, table legend, main text, or Methods section.

n/a	Confirmed	
☐	☒	The exact sample size (*n*) for each experimental group/condition, given as a discrete number and unit of measurement
☒	☐	A statement on whether measurements were taken from distinct samples or whether the same sample was measured repeatedly
☐	☒	The statistical test(s) used AND whether they are one- or two-sided<br>*Only common tests should be described solely by name; describe more complex techniques in the Methods section.*
☐	☒	A description of all covariates tested
☐	☒	A description of any assumptions or corrections, such as tests of normality and adjustment for multiple comparisons
☐	☒	A full description of the statistical parameters including central tendency (e.g. means) or other basic estimates (e.g. regression coefficient) AND variation (e.g. standard deviation) or associated estimates of uncertainty (e.g. confidence intervals)
☐	☒	For null hypothesis testing, the test statistic (e.g. *F*, *t*, *r*) with confidence intervals, effect sizes, degrees of freedom and *P* value noted<br>*Give P values as exact values whenever suitable.*
☒	☐	For Bayesian analysis, information on the choice of priors and Markov chain Monte Carlo settings
☒	☐	For hierarchical and complex designs, identification of the appropriate level for tests and full reporting of outcomes
☐	☒	Estimates of effect sizes (e.g. Cohen's *d*, Pearson's *r*), indicating how they were calculated

Our web collection on statistics for biologists contains articles on many of the points above.

Software and code

Policy information about availability of computer code

Data collection	Public data were downloaded from public repositories using wget. No software was used for data collection. Information on data used from published sources is provided under the 'data' section.
Data analysis	SAMtools v1.14; mosdepth v0.3.3; BCFtools v1.10; BCFtools v1.7; GangSTR v2.4.2(https://github.com/gymreklab/GangSTR); TRTools toolkit v4.1.0; ExpansionHunter v5.0.0; str-analysis v0.9.4 (https://github.com/broadinstitute/str-analysis); REViewer v0.2.7; BEDTools v2.27.1; BEDOPS v2.4.40; GENOVA v1.0; methylKit v1.16.1; Variant Effect Predictor v99.2 (VEP); clusterProfiler v3.18.0; GAT v1.3.4; PLINK v1.9; featureCounts v2.0.3; edgeR v3.32.1; DaPars v2.0; VCFtools v0.1.16; TissueEnrich v1.16.0; R v4.0.3. Analysis scripts for reproducing the analysis and figures in this study are provided in the GitHub repository: https://github.com/YiweiNiu/STR_2022/releases/tag/v0.1.

For manuscripts utilizing custom algorithms or software that are central to the research but not yet described in published literature, software must be made available to editors and reviewers. We strongly encourage code deposition in a community repository (e.g. GitHub). See the Nature Portfolio guidelines for submitting code & software for further information.

Data

Policy information about availability of data

All manuscripts must include a data availability statement. This statement should provide the following information, where applicable:

- Accession codes, unique identifiers, or web links for publicly available datasets
- A description of any restrictions on data availability
- For clinical datasets or third party data, please ensure that the statement adheres to our policy

The DNA sequencing data of NyuWa samples used in this study have been deposited in the Genome Sequence Archive (GSA) in National Genomics Data Center, China National Center for Bioinformation/Beijing Institute of Genomics, Chinese Academy of Sciences, under accession number HRA004185 (https://ngdc.cncb.ac.cn/gsa-human/). These data are available under restricted access for privacy protection and can be obtained by application on the GSA database website (https://ngdc.cncb.ac.cn/gsa-human/) following the guidance of "Request Data" on this website. These data have also been deposited in the National Omics Data Encyclopedia (NODE) of the Bio-Med Big Data Center, Shanghai Institute of Nutrition and Health, Chinese Academy of Sciences, under accession number OEP002803 (http://www.biosino.org/node). The user can register and login to this website and follow the guidance of "Request for Restricted Data" to request the data. A full list of pSTRs generated in this study has been deposited in the Genome Variation Map (GVM) in National Genomics Data Center, China National Center for Bioinformation/Beijing Institute of Genomics, Chinese Academy of Sciences, under accession number GVM000464 (https://ngdc.cncb.ac.cn/gvm/getProjectDetail?Project=GVM000464). The user can contact the corresponding author to apply for permission to access this data. The reference genome GRCh38 used in this study is available at https://console.cloud.google.com/storage/browser/genomics-public-data/resources/broad/hg38/v0/. The alignment files for the 1KGP dataset are available at https://ftp.1000genomes.ebi.ac.uk/vol1/ftp/data_collections/1000G_2504_high_coverage/. Genotype data for SNPs and indels for the 1KGP dataset is available at https://ftp.1000genomes.ebi.ac.uk/vol1/ftp/data_collections/1000G_2504_high_coverage/working/20201028_3202_phased/. RNA-seq data of the GEUVADIS Project is available at https://www.internationalgenome.org/data-portal/data-collection/geuvadis. The chromatin states data for GM12878, GM06990 and GM12865 cell lines is available at https://personal.broadinstitute.org/cboix/epimap/ChromHMM/observed_aux_18_hg38/CALLS/. GWAS Catalog variants are available at https://www.ebi.ac.uk/gwas/docs/file-downloads. Results of STRs in LD with GWAS SNPs, QTL analyses, and expansion analysis generated in this study are provided in the Supplementary Data file and are also available from a public website (http://bigdata.ibp.ac.cn/STR).

Human research participants

Policy information about studies involving human research participants and Sex and Gender in Research.

Reporting on sex and gender	This information has not been collected.
Population characteristics	The NyuWa genome resource was first presented in our previous work (PMID: 34788621) and has included more than 4,000 Chinese individuals to date. The details of population characteristics are available in Supplementary Data 1.
Recruitment	In our previous work (PMID: 34788621), we have presented the NyuWa genome resource, based on deep sequencing of Chinese individuals collected from hospitals and physical examination centers in China. Subsequently, more samples were collected as described in this work (PMID: 34788621), bringing the total number of samples of NyuWa dataset to more than 4,000. There is no potential self-selection bias or other biases in our work.
Ethics oversight	This study was approved by the Medical Research Ethics Committee of Institute of Biophysics, Chinese Academy of Sciences.

Note that full information on the approval of the study protocol must also be provided in the manuscript.

Field-specific reporting

Please select the one below that is the best fit for your research. If you are not sure, read the appropriate sections before making your selection.

☒ Life sciences ☐ Behavioural & social sciences ☐ Ecological, evolutionary & environmental sciences

For a reference copy of the document with all sections, see nature.com/documents/nr-reporting-summary-flat.pdf

Life sciences study design

All studies must disclose on these points even when the disclosure is negative.

Sample size	We determined the sample size according to the number of samples available from the NyuWa dataset and the 1KGP dataset. 4,013 unrelated individuals from the NyuWa dataset and 2,504 individuals from the 1KGP dataset were used in this study.
Data exclusions	No samples were excluded.
Replication	STR call replication was assessed using two different tools (GangSTR and ExpansionHunter) as described in the manuscript. We also used Mendelian inheritance rate to assess the STR call replication from parents to offsprings.
Randomization	Randomization is not relevant to this study because our study does not involve phenotype or curative effect and most analyses were performed on all available samples.

Blinding	Blinding was not performed and was not relevant in this study because we were not studying outcomes.

Reporting for specific materials, systems and methods

We require information from authors about some types of materials, experimental systems and methods used in many studies. Here, indicate whether each material, system or method listed is relevant to your study. If you are not sure if a list item applies to your research, read the appropriate section before selecting a response.

Materials & experimental systems

n/a	Involved in the study
☒	☐ Antibodies
☒	☐ Eukaryotic cell lines
☒	☐ Palaeontology and archaeology
☒	☐ Animals and other organisms
☒	☐ Clinical data
☒	☐ Dual use research of concern

Methods

n/a	Involved in the study
☒	☐ ChIP-seq
☒	☐ Flow cytometry
☒	☐ MRI-based neuroimaging
